# AI-Driven Discovery of Prototype CLEC4M Inhibitors Targeting Marburg Virus Entry via Integrated Machine Learning and Molecular Modeling

**DOI:** 10.3390/ijms27125324

**Published:** 2026-06-12

**Authors:** Mohammed Almaghrabi, Mansour S. Alturki

**Affiliations:** 1 Department of Pharmacognosy and Pharmaceutical Chemistry, College of Pharmacy, Taibah University, Al Madinah 30001, Saudi Arabia; mhmaghrabi@taibahu.edu.sa; 2 Department of Pharmaceutical Chemistry, College of Pharmacy, Imam Abdulrahman Bin Faisal University, Dammam 34212, Saudi Arabia

**Keywords:** marburg virus, CLEC4M, machine learning, phytochemicals, molecular docking, molecular dynamics simulation, density functional theory, drug discovery

## Abstract

Marburg virus (MARV), a highly pathogenic member of the Filoviridae family, causes severe hemorrhagic fever with a high case fatality rate and currently lacks effective therapeutics. The viral entry process, mediated by the interaction between the MARV glycoprotein (GP) and host receptor C-type lectin domain family 4 member M (CLEC4M) (L-SIGN), represents a critical target for early-stage intervention. The active compounds from BindingDB and the decoy from DUDE were used. The RDKit was used for feature engineering. Machine learning models were trained on an initial dataset consisting of 56 active chemicals and 1232 decoys. Among the tested algorithms, the Random Forest model demonstrated superior performance, achieving the highest discriminative ability (AUC = 0.93, MCC = 0.88) on the test set. Virtual screening of 11,032 phytochemicals resulted in 120 predicted actives, of which 42 compounds satisfied drug-likeness criteria. Subsequent molecular docking identified three lead compounds (PubChem IDs: 42608095, 5281601, and 11243993) with moderate-to-promising binding affinities (−6.3 to −6.5 kcal/mol) toward the CLEC4M binding site. ADMET analysis revealed favorable pharmacokinetic and toxicity profiles for the selected lead compounds. DFT calculations of the three compounds highlighted their electronic stability and reactive nature, indicating that PubChem IDs 42608095 and 5281601 possess particularly stable electronic properties conducive to favorable target interactions. Combining machine learning models with molecular docking and Molecular Dynamics (MD) simulations worked well in finding promising phytochemical inhibitors. The MM/GBSA binding free energy calculations further confirmed binding affinities, with values of −10.83 and −11.08 kcal/mol, respectively, suggesting favorable complex stability. These findings provide a pathway for developing new antiviral agents against MARV, pending further experimental validation and optimization.

## 1. Introduction

The Marburg virus (MARV) is a member of the Filoviridae family and is known to cause serious bleeding infections in people. Statistics show that Marburg outbreaks have been linked to close contact with infected animals, like fruit bats (which are thought to be the natural reservoir), and human-to-human transmission, usually between family members and healthcare workers in medical settings [1]. The World Health Organization (WHO) has said that MARV is a deadly zoonotic pathogen that has an 88% case fatality rate [2]. It is also thought to be a possible bioterrorism agent, and since 2015, it has been on the WHO’s Blueprint of Priority as one of the ten most important pathogens [3]. This is still the case, even though research on the topic has been going on for more than 50 years. There are many ways to treat pan-filovirus infections like MARV, including antiviral drugs, monoclonal antibodies, vaccines, cytokines, host-targeted therapeutics, and other medicines. There is no vaccine or medicine that can stop the disease caused by the Marburg virus, though [4,5].

**Figure 1:**
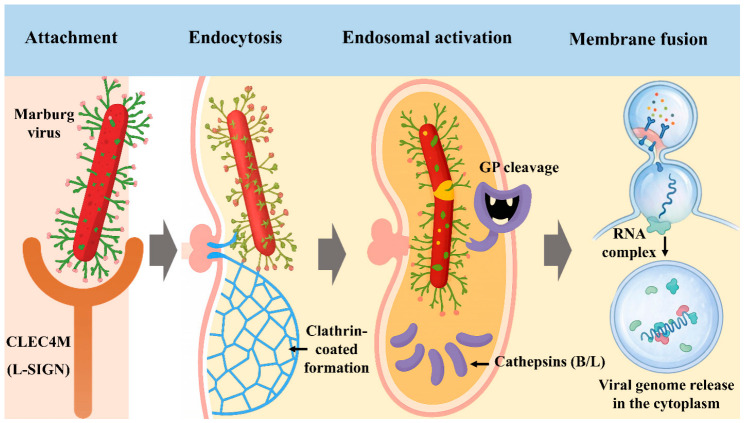
Schematic illustration of MARV entry into host cells, showing multiple stages including viral attachment to the CLEC4M (L-SIGN) receptor, clathrin-mediated endocytosis, endosomal glycoprotein (GP) cleavage by cathepsins (B/L), and membrane fusion that results in the release of the viral RNA genome into the cytoplasm.

Filoviruses only have one spike protein, which is the envelope glycoprotein (GP). It sticks to receptors and joins membranes together. Two molecules, GP1 and GP2, make up GP. These two molecules are held together by a disulfide bond. The receptor-binding domain in GP1 is what lets the virus stick to molecules on the surface of cells. The heptad-repeat regions in GP2 are what allow GP to come together to form a trimer. The internal fusion loop is thought to interact with the cell membrane [6]. MARV is a virus that has only one strand of RNA and is negative-sense. The genome codes for seven proteins, and GP is the only membrane protein. It is in charge of helping the virus attach to and enter host cells [7]. CLEC4M, also called L-SIGN or liver/lymph node-specific ICAM-3-grabbing non-integrin, is a type II transmembrane C-type lectin receptor that is mostly found on endothelial cells in the liver and lymph nodes. It has a carbohydrate recognition domain (CRD) that binds high-mannose glycans in a calcium-dependent way. This makes it easier for pathogens to be captured, endocytosed through clathrin-coated pits, and possibly trans-infected into cells that are open to infection [8]. Some pathogens have figured out how to get around CLRs’ role in activating immunity and even use CLRs for their own gain during an infection [9]. CLEC4M serves as a primary attachment factor for MARV entry, binding high-mannose glycans on GP1, thereby tethering the virus to dendritic cells, macrophages, and endothelial cells. This initiates a coordinated multi-step cascade: (i) CLEC4M-mediated attachment concentrates virions on the cell surface; (ii) clathrin-dependent endocytosis internalizes virus–receptor complexes into early endosomes; (iii) endosomal acidification drives cathepsin B/L-mediated GP1/2 cleavage and trafficking to late endosomes; and (iv) NPC1-dependent membrane fusion occurs, where cleaved GP2 engages the NPC1 cholesterol-binding domain, releasing viral genome into the cytoplasm. Reports indicate that the subversion of C-type lectin receptors (CLRs) can transform these lectins into alternative receptors or attachment factors, particularly by HIV [10], the Ebola virus [11], and the SARS-CoV virus [12]. Consequently, documented evidence suggests that various viral pathogens have independently developed strategies to utilize C-type lectin receptors as alternative receptors or attachment factors. In line with this model, MARV employs CLEC4M as an attachment factor for initial binding to host cells, particularly endothelial cells. The viral glycoprotein (GP) on the surface of MARV interacts with the CLEC4M CRD through N-linked high-mannose glycans. This helps the virus attach and makes the infection more effective, along with other lectins like DC-SIGN [13]. When GP-CLEC4M interacts, clathrin-mediated endocytosis brings MARV into cells. Endosomal cathepsins then cut GP, which exposes the fusion peptide in GP2 for membrane fusion and the release of the viral genome (Figure 1). This changes how sensitive cathepsins are and how well they can enter different MARV strains. This receptor-mediated entry elucidates MARV’s predilection for endothelial cells, compromising vascular integrity and inducing hemorrhage. Although CLEC4M facilitates filovirus attachment to the endothelium and contributes to pathogenesis, there have been no computationally designed or clinically developed small-molecule inhibitors specifically targeting CLEC4M for the Marburg virus, indicating a substantial unmet need.

While small-molecule inhibitors such as favipiravir and remdesivir have demonstrated preclinical efficacy against MARV by targeting viral RNA-dependent RNA polymerase (RdRp), yielding up to 83% survival in nonhuman primate models, their clinical translation remains limited by drawbacks including intravenous administration requirements, potential toxicity (e.g., teratogenicity for favipiravir, nephrotoxicity for remdesivir), emergence of resistance, and lack of dedicated human trials for MARV disease [1]. These replication-stage inhibitors miss early viral entry, a critical bottleneck mediated by host receptors such as CLEC4M (L-SIGN). A study by Anantpadma et al. (2016) identified nine MARV inhibitors using a pseudotyped virus-based quantitative high-throughput screening approach combined with microscopy-based image analysis [2]. Another study by M. Alsaady et al. (2023) demonstrated that in silico drug exploration found potent inhibitors of MARV targeting the VP35 protein through virtual screening and MD simulations [3].

Recent advancements in computational methodologies have revolutionized the field of drug discovery. In silico methodologies facilitate the identification and optimization of lead compounds from extensive chemical libraries, markedly decreasing the time and expenses linked to conventional experimental workflows [4,5]. Using machine learning-based methods can help learn important things that can then lead to better treatment results. Machine learning (ML)-based techniques use QSAR modeling, hit discoveries, and de novo drug design to obtain precise results. Small-molecule drugs are pivotal in drug development because their size makes it easy for them to interact with biological targets [6]. This study focuses on the identification of small drug-like molecules targeted to CLEC4M. Using the integrated approach, namely, an ML-based virtual screening approach with molecular docking and simulation to identify potential candidates, can serve as the basis for therapeutic development against MARV.

## 2. Results

### 2.1. Data Preparation, Processing, and Feature Selection

The dataset consisted of 1288 compounds in total, of which 56 were classified as active molecules sourced from BindingDB, while the remaining 1232 were inactive decoy compounds (Supplementary File S1). A total of 25 molecular features were generated using the RDKit library, and a comprehensive summary of these features is presented in Table 1.

For model development, the dataset was divided into training and testing subsets using a 70:30 ratio (Table 2). This resulted in 901 molecules being allocated to the training set (Supplementary File S2) and 387 compounds reserved for the test set (Supplementary File S3).

### 2.2. Principle Component Analysis (PCA)

PCA effectively streamlined the dataset while preserving the majority of its significant features. The first principal component (PC1) accounted for 94.6% of the variance, whereas the second principal component (PC2) accounted for 4.8%. Collectively, these two components captured 99.4% of the overall variance, as confirmed by cumulative variance analysis (Supplementary Figure S1), underscoring the most critical molecular characteristics and trends present in the data. This near-complete variance retention restricts classification to two components, as they preserve essentially all discriminative information present in the original 25-descriptor feature space. PCA loading analysis further revealed that PC1 was predominantly driven by molecular weight, with secondary contributions from NumValenceElectrons and TPSA, collectively representing the molecular size axis. PC2 was predominantly captured by TPSA, contrasted against molecular weight, reflecting a polarity axis. PCA loading heatmaps illustrating the contribution of each original descriptor to PC1 and PC2 are provided as Supplementary Materials (Supplementary Figure S2).

**Figure 2:**
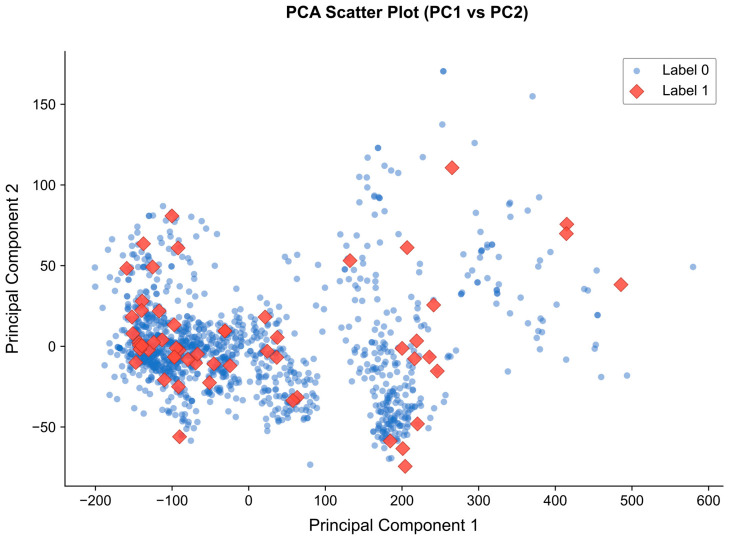
The PCA scatter plot of PC1 versus PC2 illustrates the distribution of compounds based on their principal component scores. The data points are categorized into two distinct classes, with Label 0 in blue and Label 1 in red, indicating phytochemical compounds and decoys, respectively.

Figure 2 illustrates a scatter plot of the first two principal components, distinctly differentiating the active phytochemical compounds from their inactive counterparts. This separation underscores the efficacy of the features identified through PCA in differentiating between the two groups.

### 2.3. Chemical Space and Diversity Analysis

The performance of a machine learning model largely depends on the level of chemical diversity present in the training and testing datasets. A diverse dataset improves the model’s ability to generalize, while limited variability may reduce its predictive reliability.

**Figure 3:**
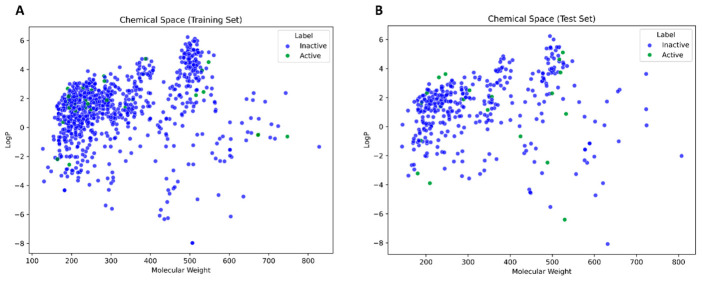
Distribution of chemical space for the training (**A**) and test (**B**) datasets illustrated using molecular weight (MW) and LogP as descriptors. Each point is color-coded, with green representing active compounds and blue indicating inactive compounds.

To assess the chemical space, a physicochemical property distribution analysis was performed for both the training and test sets using molecular weight (MW) and LogP as key descriptors (Figure 3). The MW of the compounds ranged from 127.15 to 827.89 Da, indicating the inclusion of molecules spanning a wide size spectrum. In addition, LogP values varied between −8.08 and 6.23, reflecting a broad distribution of hydrophilic and lipophilic characteristics. The comparison of training and test sets revealed similar distribution patterns across these properties, suggesting that both subsets share a comparable chemical space.

### 2.4. Machine Learning Models’ Performance Evaluation

Several supervised machine learning algorithms, namely KNN, SVM, RF, NB, and GB, were implemented using Python v3.10 to build predictive models. Their performance was assessed through multiple evaluation metrics, including accuracy, sensitivity, specificity, Matthew’s correlation coefficient (MCC), and the area under the ROC curve (AUC).

Among all models, the RF model achieved a test-set AUC of 0.9318 and MCC of 0.8798. The GB model ranked second with an AUC of 0.8437 and an MCC of 0.5683. The KNN model achieved an AUC of 0.8042 and an MCC of 0.9077, while the NB and SVM models yielded AUC values of 0.5645 and 0.4201 and MCC values of 0.5245 and 0.4000, respectively. A comprehensive comparison of these metrics is provided in Table 3.

**Figure 4:**
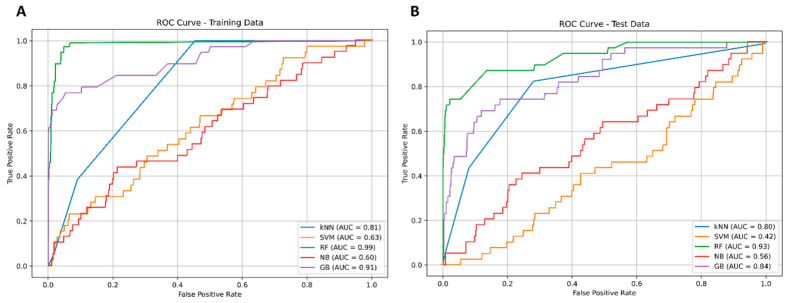
ROC-AUC for all ML models analyzed on the (**A**) training set and (**B**) test set.

To enhance the robustness of the findings, a ten-fold cross-validation was applied during model evaluation. Additionally, ROC-AUC analysis was used as a reliable indicator of classification performance. The RF model again outperformed the others, recording an AUC of 0.93, followed by GB (0.84), KNN (0.80), NB (0.56), and SVM (0.42), as depicted in Figure 4.

### 2.5. Screening of External Dataset

The Random Forest (RF) model exhibited a superior performance relative to other algorithms based on its overall superior AUC and MCC. Consequently, the RF model was chosen for the screening of phytochemicals against the CLEC4M protein. Before virtual screening, the AD of the RF model was evaluated for the complete phytochemical library (11,032 compounds) using a Williams plot (Supplementary Figure S3). A total of 9941 compounds (90.1%) fell within the defined AD, confirming that the RF model’s predictions are structurally reliable for the vast majority of the library. Out of a total of 11,032 compounds, 120 were identified as potentially active (Supplementary File S4). Subsequent evaluation was performed utilizing the Lipinski module incorporated within RDKit to compute critical physicochemical properties, including molecular weight (MW), hydrogen bond donors (HBDs), hydrogen bond acceptors (HBAs), and LogP. Among the predicted 120 active compounds, 42 met these criteria, indicating their appropriateness for further molecular docking studies and their potential as promising drug candidates (Supplementary File S5).

### 2.6. Molecular Docking and Interaction Analysis

The three-dimensional crystal structure of the CLEC4M protein was obtained from the Protein Data Bank (PDB) through the identifier “1K9J”. The 1K9J demonstrated the best overall structural quality, with a resolution of 1.90 Å, an ERRAT score of 82.5, and a VERIFY3D score of 81.01%. Ramachandran plot analysis using PROCHECK confirmed structural reliability, with 89.0% of residues in the most favored regions [A,B,L], 10.5% in additional allowed regions, 0.4% in generously allowed regions, and 0.0% in disallowed regions (Supplementary Figure S4).

A thorough library of 42 phytochemicals was subjected to screening against 1K9J, resulting in the identification of three compounds that exhibited the most favorable binding affinities among the screened compounds, which were subsequently selected for further examination. To validate the docking procedure and provide an objective reference for binding energy interpretation, remdesivir [7,8] and N-(2-{[4-(dimethylamino)phenyl]amino}-4’-methyl-4,5’-bi-1,3-thiazol-2’-yl)propenamide were included as a positive control (Table 4). N-(2-{[4-(dimethylamino)phenyl]amino}-4’-methyl-4,5’-bi-1,3-thiazol-2’-yl)propenamide is an experimentally confirmed CLEC4M inhibitor retrieved from the BindingDB database with a reported IC50 value of 1600 nM [9,10].

**Figure 5:**
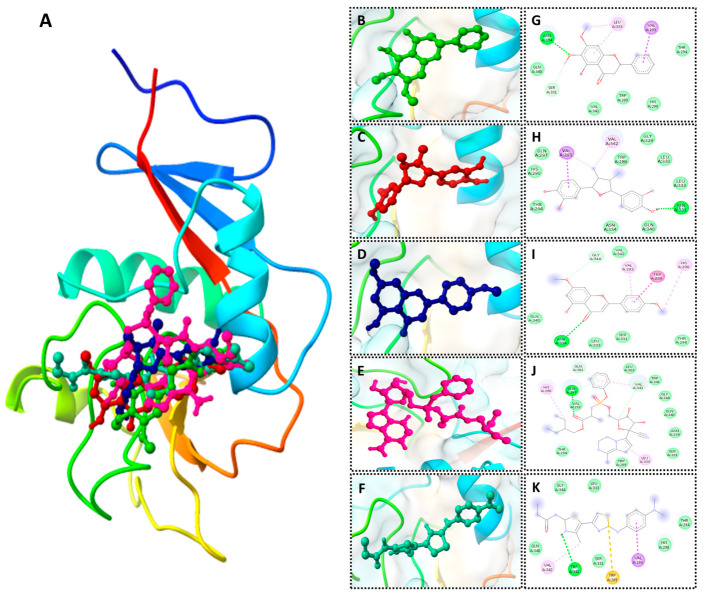
(**A**) The top three phytochemical leads and 2 positive controls, Remdesivir and N-(2-{[4-(dimethylamino)phenyl]amino}-4’-methyl-4,5’-bi-1,3-thiazol-2’-yl)propenamide, docked into the CLEC4M binding site. Three-dimensional molecular surface representation of (**B**) PubChem 42608095, (**C**) PubChem 5281601, (**D**) PubChem 11243993, (**E**) Remdesivir, (**F**) N-(2-{[4-(dimethylamino)phenyl]amino}-4’-methyl-4,5’-bi-1,3-thiazol-2’-yl)propanamide within the CLEC4M binding pocket. Two-dimensional interaction diagrams (BIOVIA Discovery Studio) of PubChem (**G**) 42608095, (**H**) 5281601, (**I**) 11243993, (**J**) Remdesivir, and (**K**) N-(2-{[4-(dimethylamino)phenyl]amino}-4’-methyl-4,5’-bi-1,3-thiazol-2’-yl)propanamide.

The phytochemical demonstrating the highest binding affinity was the complex compound 5-hydroxy-6,7-dimethoxy-2-phenyl-2,3-dihydrochromen-4-one, with a binding affinity of −6.5 and a root mean square deviation (RMSD) value of 0.77 Å. This compound exhibited notable interactions, primarily with the VAL A:293, SER A:331, LEU A:333, and ASN A:334 residues. The second compound, 4-[(2R,3R,4S,5S)-5-(3,4-dihydroxyphenyl)-3,4-dimethyloxolan-2-yl]benzene-1,2-diol, displayed a binding affinity of −6.4 and an RMSD of 0.734 Å, with interactions involving the VAL A:293, SER A:331, and VAL A:342 residues. 5-hydroxy-7-methoxy-2-(4-methoxyphenyl)chromen-4-one demonstrated a binding affinity of −6.3 and an RMSD of 1.869 Å, interacting with the TRP A:289, HIS A:290, VAL A:293, ASN A:334, and GLY A:344 residues. Remdesivir exhibited a binding affinity of −6.0 kcal/mol with an RMSD of 0.63 Å, interacting with HIS 290, GLN 297, GLN 302, LEU 333, and VAL 342. Lastly, N-(2-{[4-(dimethylamino)phenyl]amino}-4’-methyl-4,5’-bi-1,3-thiazol-2’-yl)propenamide exhibited a binding affinity of −5.9 kcal/mol with an RMSD of 2.002 Å (Figure 5). In conclusion, this detailed docking analysis elucidated specific interactions between these phytochemicals and the CLEC4M protein, highlighting their potential as novel therapeutic candidates. The compounds were further processed for ADMET and toxicity prediction.

### 2.7. ADMET Profiling and Toxicity Analysis

The ADMET evaluation of compounds with PubChem IDs 42608095, 5281601, and 11243993 indicates favorable pharmacokinetic and safety profiles, supporting their potential as drug candidates. All three showed high intestinal absorption and efficient membrane permeability, with balanced distribution and controlled CNS penetration. Metabolic analysis suggests minimal interactions with CYP enzymes, reducing the risk of drug–drug interactions, while excretion profiles are predictable. Toxicity assessments reveal no hepatotoxicity, skin sensitization, or cardiotoxicity, and overall safety margins, including LD50 and LOAEL, are acceptable (Table 5). Importantly, all three candidate compounds were predicted to be non-inhibitors of hERG I, suggesting the absence of a strong cardiotoxicity risk associated with severe cardiac ion channel blockage. However, PubChem 5281601 and 11243993 were predicted as hERG II inhibitors; hERG II predictions alone are generally considered less alarming than strong hERG I inhibition and, therefore, may indicate only a comparatively lower potential for cardiotoxic liability. These three compounds were selected for further DFT analysis to study their electronic structure, reactivity, and stability.

### 2.8. DFT Analysis

**Figure 6:**
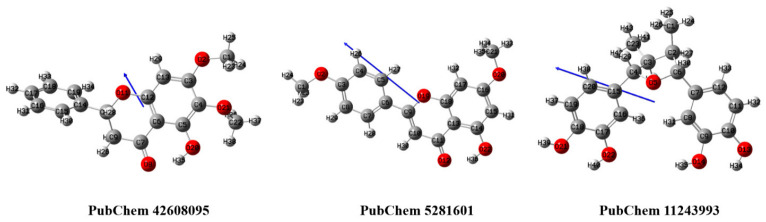
Optimized structures of the lead compounds (PubChem 42608095, PubChem 5281601, and PubChem 11243993), where carbon, hydrogen, and oxygen atoms are color-coded in gray, white, and red, respectively. The blue arrows represent the direction and relative magnitude of the total molecular dipole moment vectors.

The stability of the structure of the chosen compounds, their electronic properties, and their chemical reactivity were studied by using Density Functional Theory calculations. The optimized molecular structures, frontier molecular orbital distributions, and electrostatic potential maps can give a lot of information about the physicochemical behavior of the compounds. The optimized structures, orbital distributions and electrostatic potential maps of the molecules are shown in Figure 6, while calculated parameters such as HOMO, LUMO, energy difference, dipole moment, and global reactivity descriptors are summarized in Table 6. The frontier molecular orbital energies were converted from Hartree to electron volts (eV) using the conversion factor (1 Hartree = 27.2114 eV) to be consistent with data from the literature. It should be noted that the DFT calculations did not aim to directly predict antiviral activity, but rather to compare the intrinsic electronic properties of structurally related lead compounds. The descriptors calculated should, therefore, be considered as complementary physicochemical parameters that might affect molecular recognition and binding properties and should not be used as a biological efficacy ‘fingerprint’ alone.

The DFT calculations were carried out at the B3LYP functional and 6-311G basis set. This method is suitable for predicting relative trends in electronic properties for structurally related compounds. While solvent effects were not taken into account, the descriptors obtained are adequate for the qualitative analysis of reactivity. This could be extended to include an implicit solvent model (such as PCM or SMD) in future studies to improve the accuracy of reactivity assessments in biological systems.

#### 2.8.1. Optimized Structures

The optimized molecular geometry of the examined compounds is shown in Figure 6. Geometry optimization offers the most stable geometrical configuration of each molecule and assists in comprehending how they are structurally oriented and the distribution of electrons. The results of the optimization indicate that the three molecules had stabilized conformations with low electronic energies. Of the molecules under study, PubChem 42608095 was found to have an electronic energy of −1033.498346 Hartree, whereas PubChem 5281601 had an electronic energy of −1032.308641 Hartree, which is highly structurally stable. Comparatively, PubChem 11243993 had an electronic energy of −1074.029739 Hartree, but stability was not the determinant of chemical reactivity, and for this reason, additional electronic work was carried out [11]. For the purpose of investigating the possible correlation between the electronic properties and protein-binding ability, the DFT descriptors were compared qualitatively to molecular docking results. Compounds with smaller HOMO–LUMO gaps and high softness can have a better capacity for charge redistribution and, thus, can promote intermolecular interaction. The present study revealed PubChem 42608095 and PubChem 5281601 as both having good docking scores, as well as a comparatively smaller energy gap. This observation, however, should not be taken as an actual biological boost, but rather a correlation for enhanced biological activity, which must be tested in an experimental setting.

The values of the dipole moment also give information on the polarity of the molecules and intermolecular interactions. The highest dipole moments were found in PubChem 5281601 (7.549542 Debye), PubChem 11243993 (6.043968 Debye), and PubChem 42608095 (1.613618 Debye). An increased dipole moment would tend to increase the interactions of the molecule with the biological target because of improved electrostatic interactions. Therefore, PubChem 5281601 has a high level of polarity, which makes it likely that it will interact better with molecular recognition and binding.

#### 2.8.2. Frontier Molecular Orbital Analysis (FMO)

**Figure 7:**
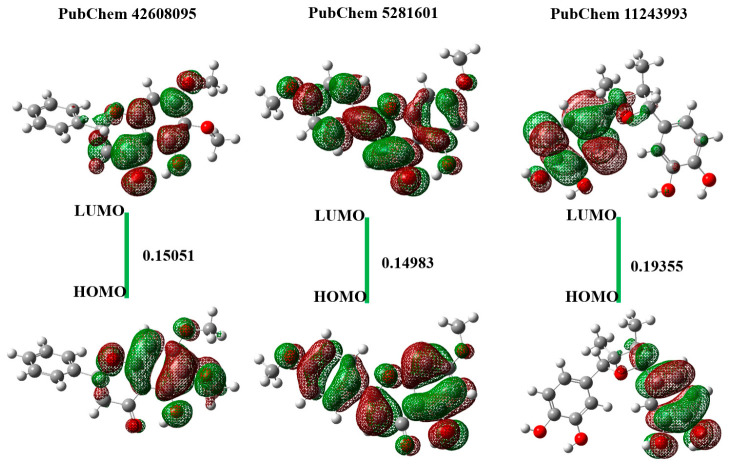
HOMO, LUMO, and energy gap of lead compounds. The green and red mesh regions represent the positive and negative phases of the molecular orbital wavefunctions, respectively.

The application of Frontier Molecular Orbital analysis is very important in the study of molecular reactivity, charge transfer capability, and chemical stability. Figure 7 and Table 1 demonstrate the distribution of HOMOs and LUMOs of the examined compounds and energy values, respectively. HOMO is the donation ability of a molecule by an electron, and LUMO is its ability to accept an electron. Another crucial parameter that shows the stability of a molecule and chemical reactivity is the energy difference between these orbitals, which is often referred to as the HOMO and LUMO energy gap [12].

PubChem 42608095, PubChem 5281601, and PubChem 11243993 had calculated HOMO energies of 6.123 eV, −6.275 eV, and −5.754 eV, respectively. Equally, the LUMO energies were found to be −2.028 eV, −2.196 eV, and −0.485eV of the respective compounds. The HOMO–LUMO energy gaps of PubChem 42608095, PubChem 5281601, and PubChem 11243993 were determined to be 4.095 eV, 4.075 eV, and 5.269 eV, respectively. The decrease in the size of the energy gaps between PubChem 42608095 and PubChem 5281601 is a feature that suggests that the chemical reactivity and charge transfer ability are better in PubChem 42608095 and PubChem 5281601 than in PubChem 11243993. Thus, the two compounds show increased electronic flexibility and are most likely to have stronger interactions with biological targets.

#### 2.8.3. Global Chemical Reactivity Descriptors

Global reactivity descriptors offer additional information on the chemical behavior, stability, and potential of interaction of molecules. The values of HOMO and LUMO energies obtained under the Koopmans theorem were used to compute these descriptors, and are listed in Table 6. The ionization potential (I) values were estimated at 6.123 eV, 6.275 eV, and 5.754 eV in PubChem 42608095, PubChem 5281601, and PubChem 11243993, respectively. It was also found that the electron affinity (A) was 2.028 eV, 2.196 eV, and 0.485 eV. These findings imply that PubChem 5281601 has the largest electron affinity, meaning that it is more likely to accept electrons during molecular interactions.

The values of chemical hardness (η) were 2.047 eV, 2.039 eV, and 2.634 eV, respectively. Reduced hardness implies greater chemical reactivity, and again justifies the greater chemical reactivity of PubChem 42608095 and PubChem 5281601. Chemical softness (S) values were found to be 0.489 eV^−1^, 0.491 eV^−1^, and 0.380 eV^−1^. An increase in the value of softness is associated with an increase in polarizability and interaction ability. To this end, the greater values of softness of PubChem 42608095 and PubChem 5281601 only substantiate the fact that they are more chemically reactive than PubChem 11243993. The values of the electrophilicity indices, 16.23 eV, 17.61 eV and 7.39 eV, showed that PubChem 5281601 is the most electrophilic substance, followed by PubChem 42608095. These findings show the better electronic characteristics of the first two compounds.

#### 2.8.4. Molecular Electrostatic Potential (MEP) Analysis

**Figure 8:**
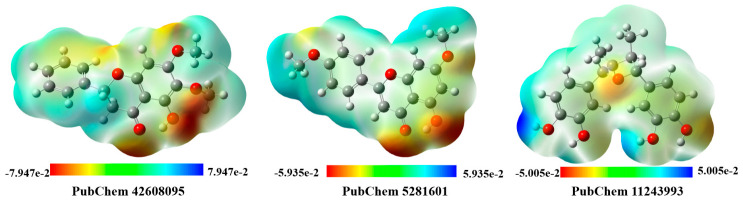
MEP structure and scale of lead compounds.

The Molecular Electrostatic Potential (MEP) surfaces show valuable information on the distribution of charge on the molecular surface and contribute to the location of potential areas of electrophilic and nucleophilic attacks. Figure 8 shows the MEP maps of the compounds under study. Regions in the MEP maps that are red are those that are rich in electrons, and, thus, those areas are selectable for electrophilic attacks, whereas regions that are blue are those that are deficient in electrons and, thus, are susceptible to nucleophilic attack. The green color is normally used to symbolize the neutral regions.

The MEP analysis showed that electronegative elements, including oxygen and nitrogen, form highly negative potentials in PubChem 42608095 and PubChem 5281601, and it can be argued that the electronegativity of oxygen and nitrogen elements is strongly localized throughout the atoms. Such electron-rich areas can enhance more intense intermolecular interactions with receptor active sites. Conversely, PubChem 11243993 had a relatively smaller electrostatic potential distribution, which is a sign of weaker charge localization and, accordingly, fewer interaction possibilities. Thus, the MEP analysis also contributes to the excellent electronic properties of PubChem 42608095 and PubChem 5281601.

On the whole, the analysis of the Density Functional Theory indicated that the electronic behavior of the examined compounds is substantially different. The reduced HOMO–LUMO energy gap values, increased softness values, and positive electrophilicity index values of PubChem 42608095 and PubChem 5281601 are signs of increased chemical reactivity and better electron transfer behavior. Further, their positive distributions of electrostatic potentials and values of the dipole moment indicate a higher potential of interaction with the biological targets.

By contrast, PubChem 11243993 exhibited a higher energy gap, decreased softness, and reduced electrophilic character, which suggests a relatively reduced reactivity. According to the overall outcome of structural optimization, frontier molecular orbital analysis, global reactivity descriptors, and electrostatic potential mapping, PubChem 42608095 and PubChem 5281601 are possibly superior lead compounds in the current investigation, where they exhibit better electronic and physicochemical characteristics. It should be noted that the current DFT calculations were performed in the gas phase and may not account for the solvent effects in biological systems. However, the comparative analysis of electronic descriptors for the compounds is valid and offers insights into their relative reactivities. Future studies should include solvent models to improve the predictive power of these analyses.

### 2.9. Molecular Dynamics Simulations (MDSs)

**Figure 9:**
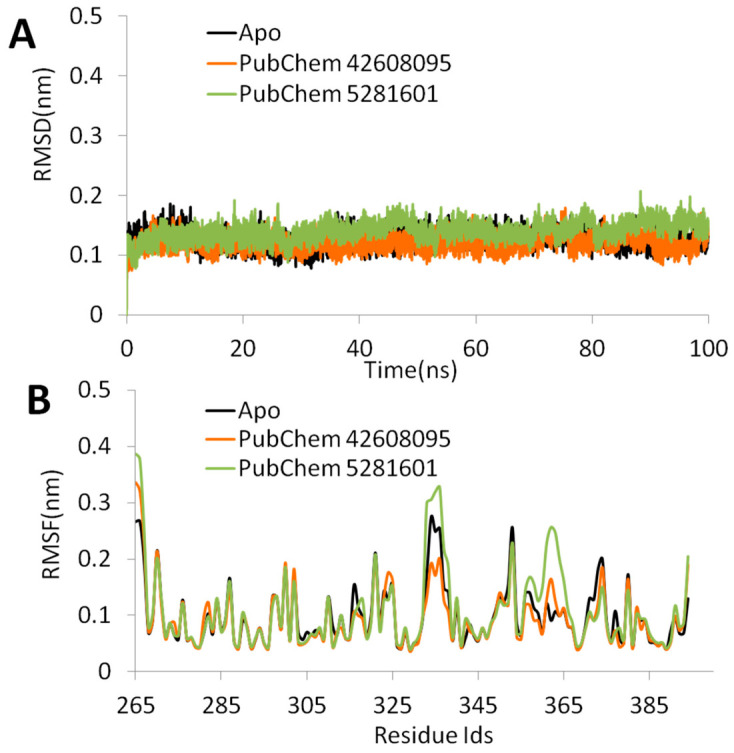
(**A**) RMSD profile of the simulated complexes and the Apo form of the target protein. (**B**) Residue-level RMSF profile of the simulated complexes and the Apo form of the target protein.

**Figure 10:**
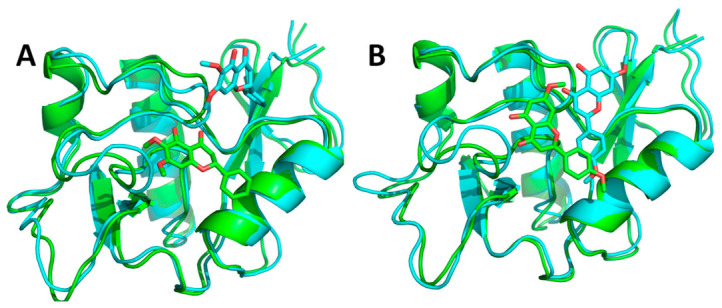
Superimposition of 0 ns and 100 ns frames of MDS trajectories, illustrating structural stability. (**A**) PubChem 42608095, and (**B**) PubChem 5281601, illustrating structural stability, where the initial (0 ns) and final (100 ns) conformations are color-coded in green and cyan, respectively.

MDSs were performed to assess the stability of the complexes, and the trajectories were examined using different matrices, including root mean square deviation (RMSD) and root mean square fluctuations (RMSFs). The RMSD analysis showed that both complexes were rather stable throughout the simulation (Figure 9A). The RMSD values for all simulated systems were less than 0.2 nm, showing that the protein structure remained conformationally stable throughout the simulations. Furthermore, to evaluate the overall structural stability and binding mode conservation of the complexes during the simulation period, the initial and final trajectory frames were extracted and superimposed. The superimposition analysis demonstrated minimal conformational deviation between the starting and ending structures, further supporting the stability of the protein–ligand complexes throughout the MD simulation (Figure 10).

Further, the RMSF analysis was used to investigate the flexibility of the residues within the protein structure (Figure 9B). The majority of residues showed low fluctuation values, indicating that the protein had little flexibility. Most regions of the protein exhibited an RMSF below 0.2 nm, except in a few loop regions, which are typically more flexible. Overall, the MD simulation results indicate that both ligands form stable complexes with the target protein, maintaining structural integrity throughout the simulation.

#### 2.9.1. Radius of Gyration (Rg) and Solvent-Accessible Surface Area (SASA)

**Figure 11:**
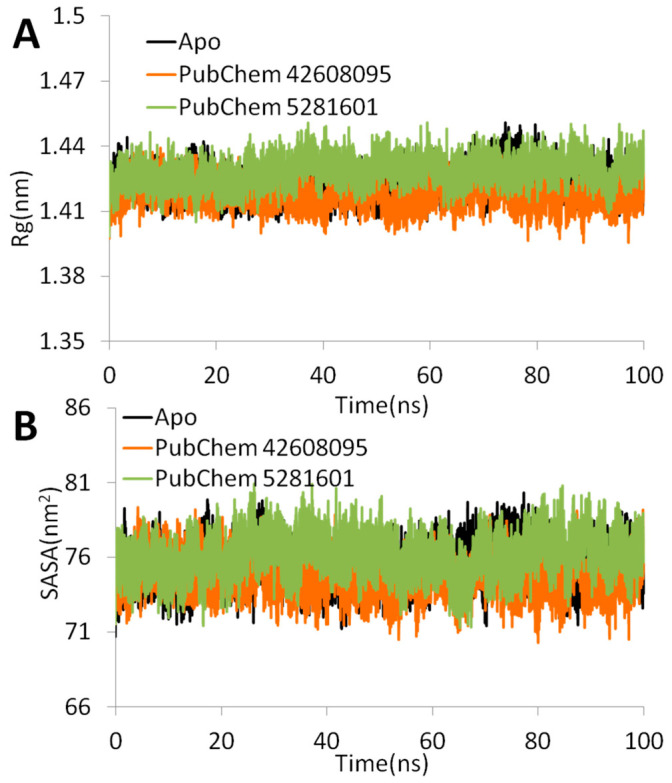
(**A**) Rg of the simulated complexes and the Apo form of the target protein. (**B**) SASA profile of the simulated complexes and the Apo form of the target protein.

In order to analyze the stability and folding behavior of the protein, Rg and SASA analyses were performed on the simulated trajectories. The Apo form and the complex with PubChem had similar Rg values, which fluctuated around 1.43 nm (Figure 11A). However, binding of PubChem 42608095 produced a somewhat lower and more consistent Rg value (about 1.41 nm), indicating that this ligand generates a more compact structure conformation. Consistent with the Rg analysis, the SASA analysis (Figure 11B) revealed that all systems had a consistent surface area throughout the simulation. The SASA values for the PubChem 42608095 complex remained slightly lower than those for the other systems, showing that the protein residues were exposed to less solvent, which is often a sign of increased structural stability and tighter packing after ligand binding. No notable unfolding events were recorded, indicating that all complexes remained structurally intact during the production run.

#### 2.9.2. Hydrogen Bond Analysis

**Figure 12:**
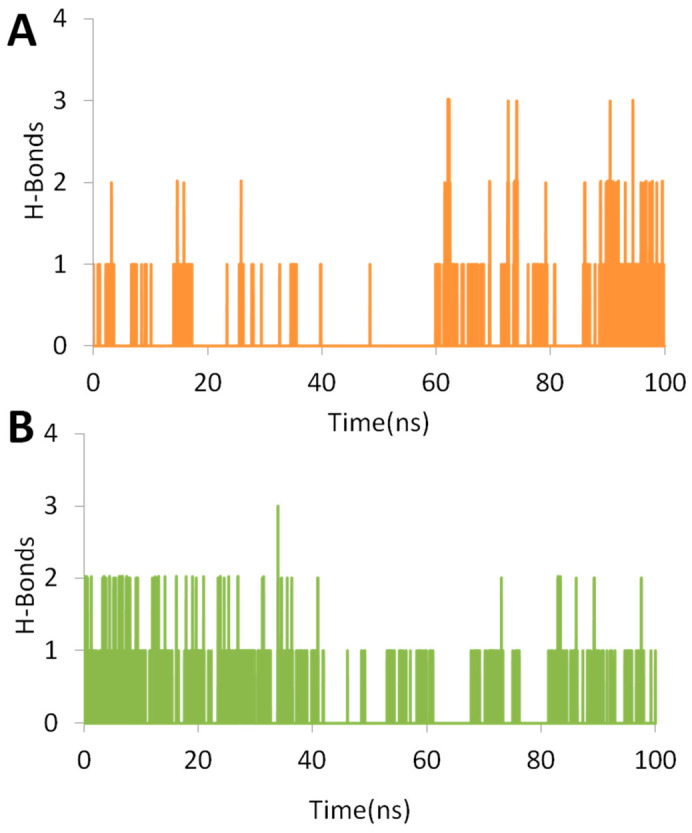
Hydrogen bond formation of the compounds with the target protein over a time of 100 ns MDS. (**A**) PubChem 42608095, (**B**) PubChem 528160.

The formation of hydrogen bonds between proteins and compounds was analyzed to assess the stability of intermolecular interactions. The results show that both compounds maintained hydrogen bond interactions throughout the simulation time. In complex PubChem 42608095, the number of hydrogen bonds fluctuated during the simulation, with hydrogen bonds up to two, suggesting transient but recurring stabilization between the ligand and the binding site residues (Figure 12A). Similarly, PubChem 5281601 formed up to two hydrogen bonds consistently during the simulation period (Figure 12B). The occurrence of constant hydrogen bonding suggests that the ligand was appropriately orientated within the binding pocket throughout the simulation.

#### 2.9.3. Principal Component Analysis (PCA)

**Figure 13:**
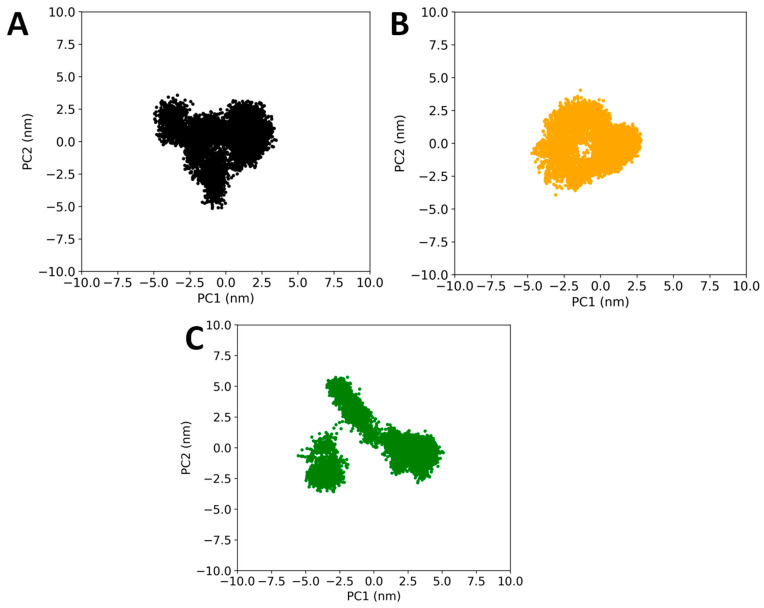
Principal Component Analysis of the (**A**) Apo, (**B**) PubChem 42608095, (**C**) PubChem 5281601.

In order to assess the essential dynamics of the protein, PCA was performed on the apo and ligand-bound states. This analysis represents the overall motion and conformational space of the protein structure. In the apo state, the protein structure exhibited broad distribution with three distinct clusters, suggesting protein structure flexibility (Figure 13A). Upon binding with PubChem 42608095, the conformational space became compact and more localized, suggesting that Ligand 1 binding stabilizes the protein and restricts its global motions (Figure 13B). In contrast, PubChem 5281601 displayed a distinct distribution as compared to apo and PubChem 42608095, indicating a different mode of conformational transition or specific flexibility induced by this ligand (Figure 13C). Overall, the PCA results confirm that PubChem 42608095 induces the highest structural stability among the tested systems.

### 2.10. MM-GBSA Analysis

The binding free energy of complexes was further assessed using the MM/GBSA approach to measure the energetic contributions to complex stability (Table 7). The results show that both compounds exhibited favorable binding with the target protein. In PubChem 42608095, the binding was mostly driven by van der Waals and electrostatic interactions. The total binding energy (ΔTOTAL) was −10.83 kcal/mol, suggesting a thermodynamically favorable interaction. Similarly, PubChem 5281601 showed stronger van der Waals and electrostatic energy contributions. This molecule had higher favorable van der Waals and electrostatic interaction energies, resulting in a negative gas-phase energy (ΔGGAS). The binding free energy (ΔTOTAL) for this compound was −11.08 kcal/mol.

## 3. Discussion

Marburg virus (MARV), a highly virulent member of the Filoviridae family, remains a significant global health threat due to its high case fatality rate and the lack of approved treatments. Increasing evidence indicates that, similar to other filoviruses [13], MARV uses multiple host cell factors to ensure successful entry and infection. Among these, the C-type lectin receptor CLEC4M has become a key attachment factor that boosts viral binding to endothelial cells through interactions with high-mannose glycans on the viral GP [14]. This interaction promotes clathrin-mediated endocytosis and subsequent viral internalization, underscoring the importance of host–virus interactions in disease development. At the same time, endosomal receptor NPC1 serves as the obligatory fusion trigger post-cathepsin priming. Unlike NPC1, which lacks tractable small-molecule pockets due to its intracellular inaccessibility, CLEC4M offers a druggable extracellular domain with established lectin inhibitor precedents. Additionally, previous studies have shown that filoviruses utilize a complex network of host pathways and receptors during entry, including lectins, endosomal proteases, and signaling molecules [15]. Notably, host signaling systems such as G protein-coupled receptors have been linked either directly or indirectly to facilitating viral entry, suggesting that MARV infection depends on the coordinated involvement of multiple host factors rather than a single receptor [16]. Unlike other host factors that have been extensively studied, CLEC4M has not been specifically exploited for the design of small-molecule inhibitor against MARV. Inhibiting human CLEC4M protein activity may help reduce viral entry and pathogenesis. While CLEC4M is minimally expressed in hepatocytes and systemic endothelium, primary hemorrhagic disease sites and alternative attachment pathways (heparan sulfate, Axl) provide functional redundancy, allowing viral escape. Consequently, CLEC4M monotherapy would provide only partial protection against systemic infection. The therapeutic potential would be substantially enhanced through combination strategies simultaneously targeting multiple entry steps, such as combining CLEC4M inhibitors with downstream obligatory factors (NPC1, cathepsin processing) or alternative attachment pathways [17,18].

A range of methods, including ML-based screening, molecular docking, MM-GBSA, MD simulation, and DFT, were employed to identify potential drug candidates. The reliability of any ML-based drug discovery approach largely depends on the quality and diversity of the dataset. In this study, a curated dataset of 1288 compounds, comprising a limited number of active molecules and a larger pool of decoys, was utilized. The SMILES notation is used for feature extraction. Despite the class imbalance, the molecular descriptors generated by the RDKit library provided a comprehensive representation of the physicochemical characteristics of compounds. After that, the dataset was split into 70% training and 30% for testing to validate the model [19].

Molecular docking provides insights into molecular-level interactions by evaluating site-specific binding affinity between target proteins and compounds [20]. Thus, 42 phytochemicals were subjected to screening against the target protein, resulting in the identification of three compounds that exhibited the most favorable binding affinities among the screened compounds with the CLEC4M protein during molecular docking. The three identified compounds, 5-hydroxy-6,7-dimethoxy-2-phenyl-2,3-dihydrochromen-4-one, 4-[(2R,3R,4S,5S)-5-(3,4-dihydroxyphenyl)-3,4-dimethyloxolan-2-yl] benzene-1,2-diol, and 5-hydroxy-7-methoxy-2-(4-methoxyphenyl) chromen-4-one, formed significant numbers of stable interactions with key residues such as VAL293, SER331, and ASN334, which are likely important for ligand recognition and receptor function with the CLEC4M protein (Table 4). A previous study reported that the 5-hydroxy-6,7-dimethoxy-2-phenyl-2,3-dihydrochromen-4-one has exhibited strong binding affinity against the monkeypox virus [21], which supports our findings. Importantly, the binding pocket identified via CASTp and used for molecular docking corresponds to the Carbohydrate Recognition Domain (CRD) of CLEC4M, the biologically relevant site for calcium-dependent binding of N-linked high-mannose glycans present on MARV GP1. Key interacting residues identified in our docking analysis, including TRP 289, HIS 290, ASN 334, and VAL 293, are located within this CRD region. Residues such as ASN 334 and HIS 290 are positioned at or near the calcium coordination and glycan-binding sub-site characteristic of C-type lectin CRDs, while TRP 289 represents a conserved aromatic residue known to contribute to hydrophobic stacking interactions with sugar moieties. Disruption of ligand engagement at these residues, therefore, has the potential to directly interfere with the CLEC4M–GP1 interaction that initiates MARV attachment and subsequent clathrin-mediated endocytosis [22,23]. The ADMET profiling of these compounds with PubChem IDs 42608095, 5281601, and 11243993 indicates favorable pharmacokinetic and safety profiles, supporting their potential as drug candidates (Table 5).

The HOMO–LUMO energy gap, electrophilicity, hardness, and softness are descriptors that describe the intrinsic electronic properties of isolated molecules and are not directly linked to the effects of solvent, protein flexibility, or the complexity of the biological environment. Hence, the descriptors were applied in this study as additional physicochemical parameters, supporting docking and molecular dynamics studies, not as standalone predictors of antiviral activity. The lower HOMO–LUMO energy gaps observed for key compounds indicate higher chemical reactivity and better interaction potential with the target protein [24]. Additionally, molecular electrostatic potential analysis revealed electron-rich regions that may facilitate stronger binding interactions with CLEC4M active site residues. These electronic characteristics could further support the suitability of the identified compounds as promising drug candidates [25]. This study utilized a 100 ns molecular dynamics simulation of the principal compounds to assess the structural stability of the ligand–macromolecule complex within the biological system. Parameters, including RMSD, RMSF, Rg, and SASA, were analyzed to provide a clearer picture of the stability of the complexes. The low RMSD and RMSF values observed throughout the simulation indicate that the complexes remained structurally stable, with minimal conformational fluctuations. Additional parameters such as Rg and SASA further confirmed that ligand binding did not destabilize the protein structure; rather, in some cases, it enhanced compactness and stability. Consistent hydrogen-bond interactions throughout the simulation highlight the persistence of key intermolecular interactions, thereby reinforcing the reliability of docking predictions [26]. Changes in Rg trajectories during the simulation show that either the protein was folding into a more stable state or it was unfolding because its shape was changing. A decreasing Rg trend suggests that proteins are folding into a stable shape, while an increasing trend means that they are unfolding or denaturing [27]. Furthermore, binding free energy calculations using the MM/GBSA approach revealed that the selected lead compounds exhibit favorable thermodynamic profiles, with significant contributions from van der Waals and electrostatic interactions. The top compounds, 42608095 and 5281601, exhibit −10.83 kcal/mol and −11.08 kcal/mol binding energies, respectively. These findings are consistent with previous computational studies, in which lower binding free energies correlate with greater binding stability and inhibitory potential [28].

PCA uses big datasets to find important biological patterns that show how proteins change shape. It lowers the number of dimensions by showing atomic coordinates from MD trajectories as principal components (PCs). This makes it possible to see and understand global motions instead of looking at individual atomic fluctuations [29]. Dimensionality reduction through PCA further enhanced the dataset’s robustness by capturing 99.4% of the total variance in the first two principal components. The findings are consistent with the research conducted by Alshehri, F. F. (2023), which employed 56 active chemicals and 1801 decoys to compute 33 features [30]. But in Samad, A. et al., a total of 206 features were computed [31]. This high variance coverage indicates that the selected molecular features effectively describe the dataset and are sufficient for distinguishing active compounds from inactive ones. Multiple machine learning algorithms were evaluated to ensure optimal model selection, including KNN, SVM, NB, GB, and RF. Among these, the RF model demonstrated a superior predictive performance, achieving high accuracy, MCC, and AUC values. The robustness of the RF model can be attributed to its ensemble nature, which allows it to handle nonlinear relationships and complex feature interactions more effectively than traditional models. Another study identified the RF model as the best-performing approach and utilized it to screen a library of 9000 phytochemicals for the prediction of active molecules [32].

As with any computational investigation, the findings of this study should be interpreted in light of certain inherent limitations. SMOTE-based oversampling, while effective at addressing class imbalance, generates synthetic minority-class samples by interpolating between existing active compounds rather than introducing genuinely novel chemical entities, which may introduce a degree of interpolation bias in model performance metrics. Nevertheless, comparable published studies have employed similarly limited numbers of experimentally confirmed active compounds for ML-based virtual screening. For instance, Alshehri (2023) utilized 56 active compounds [30], Samad et al. (2023) utilized 101 active compounds [31], and Almatroudi (2024) utilized 226 active compounds [33], demonstrating that such approaches remain scientifically valid when supported by appropriate oversampling strategies such as SMOTE and rigorous model evaluation. Furthermore, CLEC4M functions physiologically as a higher-order multimer stabilized by the neck domain; the monomeric CRD-based docking approach adopted here may not fully recapitulate cooperative multivalent binding effects present in the native quaternary context. Finally, all findings are derived exclusively from in silico analyses, and experimental validation through in vitro and in vivo studies remains essential to confirm the binding affinity, biological activity, and therapeutic potential of the identified lead candidates. Nevertheless, integrating machine learning, molecular docking, molecular dynamics, and quantum chemical approaches represents a promising strategy for targeting CLEC4M as a host receptor, potentially circumventing resistance arising from viral mutations.

## 4. Materials and Methods

### 4.1. Dataset Curation and Preprocessing

**Figure 14:**
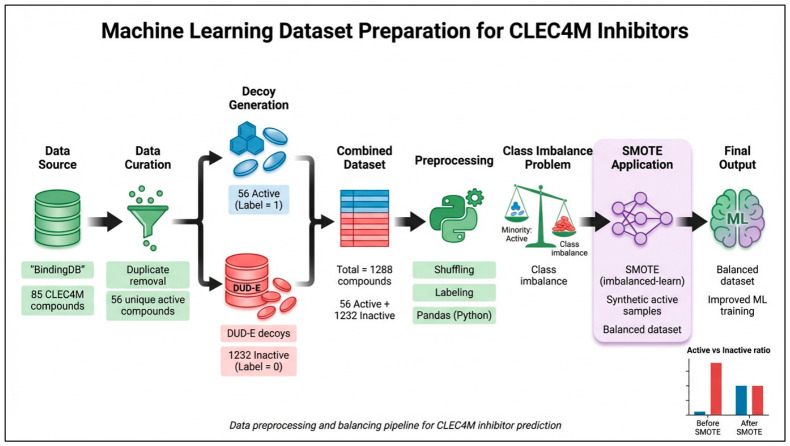
Dataset preparation workflow for CLEC4M inhibitor classification. Eighty-five compounds were retrieved from BindingDB and curated to obtain 56 unique active molecules. A total of 1232 decoys were generated from DUD-E as inactive samples. The combined dataset (1288 compounds) was labeled, shuffled, and processed using Python. Class imbalance was addressed using SMOTE to generate synthetic active samples and improve model performance.

A total of 85 compounds targeting CLEC4M were initially retrieved from the BindingDB database (Figure 14) [34]. During the curation stage, duplicate entries were identified and removed to ensure dataset quality and avoid redundancy. After duplicate removal, 56 unique active compounds remained and were considered as experimentally reported CLEC4M inhibitors. To construct a balanced machine learning dataset, decoy molecules representing inactive compounds were generated using the DUD-E database [35]. The generated decoys were subsequently screened to remove duplicate structures, resulting in 1232 unique decoy molecules, which were retained as inactive samples for the study. Following dataset preparation, a combined dataset of 1288 compounds was obtained, consisting of 56 active compounds and 1232 inactive decoys. For supervised learning, the active molecules were assigned the label “1”, whereas the decoy molecules were labeled “0” [31]. Before model development, the dataset was randomly shuffled to ensure an even distribution of active and inactive compounds and to eliminate any ordering bias that could influence model training. Because the dataset contained a significant class imbalance between active and inactive compounds, the Synthetic Minority Over-sampling Technique (SMOTE) was applied using the imbalanced-learn package (https://imbalanced-learn.org/) [36] to generate synthetic samples of the minority class [37]. This approach helps balance the dataset by interpolating new samples from existing active molecules, thereby improving the model’s ability to learn patterns associated with active compounds while reducing bias toward the majority class [38]. All dataset preprocessing steps, including duplicate removal, dataset shuffling, and labeling, were performed using the Pandas library in Python v3.10 [39].

### 4.2. Feature Engineering, Molecular Descriptor Generation, and Dataset Partitioning

To numerically represent the chemical structures for machine learning analysis, 25 molecular descriptors were calculated for each compound using the RDKit library in Python [40]. The descriptors were generated from the SMILES representation of each molecule and included physicochemical properties (MolWt, MolLogP, TPSA), electronic properties (MaxPartial-Charge, MinPartialCharge, MaxEStateIndex, MinEStateIndex), topological features (Chi0, Chi3n, BalabanJ, FpDensityMorgan1), and structural descriptors (RingCount, Nu-mAromaticRings, NumSaturatedRings, NumAliphaticCarbocycles, NumAromaticCarbo-cycles, NumHeteroatoms, NumRotatableBonds, NumAtoms, NumHeavyAtoms, Frac-tionCSP3, NumHAcceptors, NumHDonors, qed, NumValenceElectrons). Partial atomic charges were calculated using the Gasteiger charge method implemented in RDKit [41]. Numerical computations and handling of missing values were performed using the NumPy package v1.26 [42]. Data manipulation and descriptor organization were carried out using the Pandas library v2.2 [43]. To further explore the descriptor distribution within the dataset, summary statistical measures including mean, median, mode, maximum, minimum, and standard deviation were calculated using Pandas and the SciPy package v1.13 [44]. Finally, the prepared dataset was divided into training and testing subsets using a 70:30 ratio to enable model development and independent evaluation. The data splitting was performed using the train_test_split function from the scikit-learn library v1.4 [45]. SMOTE was applied exclusively with k_neighbors = 5 and random_state = 1, resampling the active class to match the inactive class distribution in the training set.

### 4.3. Chemical Space Analysis, Diversity Assessment, and Principal Component Analysis (PCA)

To explore the distribution of compounds within the dataset, chemical space analysis was performed using two key physicochemical descriptors: molecular weight (MolWt) and logarithm of the octanol–water partition coefficient (MolLogP). These descriptors provide a general representation of molecular size and hydrophobicity, which are important characteristics influencing ligand–target interactions [46]. Scatter plots were generated using Seaborn v0.13 and Matplotlib v3.8 to visualize the distributions of active and inactive compounds in both the training and testing datasets.

Following chemical space exploration, Principal Component Analysis (PCA) was applied to the calculated molecular descriptors to reduce the dataset’s dimensionality while retaining the most significant variance. PCA was first performed to evaluate the variance explained by each component, and the first two principal components (PC1 and PC2) were selected for visualization. A two-dimensional scatter plot was generated to illustrate the distribution of active and inactive compounds in the reduced feature space [47,48].

### 4.4. Machine Learning Model Development

To develop predictive models for the classification of active and inactive compounds, five supervised machine learning algorithms were employed: k-nearest neighbors (kNN) [49], support vector machine (SVM) [50], Random Forest (RF) [51], Gaussian naïve Bayes (NB) [52], and gradient boosting (GB) [53]. Model implementation was carried out using the scikit-learn library in Python. The PCA-transformed dataset, consisting of the first two principal components and class labels, was used as input for model training and evaluation.

The dataset was divided into training and testing subsets using a 70:30 stratified split to preserve the class distribution between active and inactive compounds. For models sensitive to feature scaling, including kNN and SVM, a pipeline incorporating StandardScaler was applied before classification. Hyperparameter optimization was performed using GridSearchCV with 10-fold cross-validation and ROC-AUC as the scoring criterion [54]. For the kNN model, the number of neighbors was evaluated at 5, 10, and 15 with both uniform and distance-based weighting schemes [55]. The SVM model was implemented with an RBF kernel, while the parameters C and gamma were optimized [56]. The RF model was developed using balanced subsampling, and its parameters, including the number of trees, maximum depth, minimum samples required for split and leaf nodes, and maximum feature selection, were tuned [57]. The GB model was optimized by adjusting the number of estimators, learning rate, and tree depth [58].

### 4.5. Cross-Validation and Performance Evaluation

To evaluate the robustness and predictive performance of the developed machine learning models, 10-fold cross-validation was employed during the training process. In this approach, the training dataset was divided into ten subsets, where nine subsets were used for model training and the remaining subset was used for validation. This procedure was repeated iteratively so that each subset served as a validation set once, allowing a reliable estimation of the model’s generalization performance.

The predictive performance of the models was assessed on both the training and independent test datasets using several statistical evaluation metrics, including accuracy, sensitivity (recall), specificity, Matthews correlation coefficient (MCC), and the area under the receiver operating characteristic curve (AUC) [59,60,61]. These metrics provide a comprehensive evaluation of classification performance, particularly when dealing with imbalanced datasets.

### 4.6. Model Serialization and Virtual Screening, and Applicability Domain (AD) Analysis of an External Dataset

After model development and evaluation, the trained machine learning models were preserved through model serialization to enable their reuse for external predictions. Serialization was performed using the pickle module in Python, which allows trained models to be saved as binary files and later reloaded without the need for retraining. The finalized classifiers, including kNN, SVM, RF, NB, and GB, were trained using the PCA-transformed feature set and subsequently stored as serialized model files for downstream applications.

The predictive capability of the trained models was then applied to virtual screening of an external dataset consisting of 11,032 phytochemical compounds derived from medicinal plants. These compounds represented molecules with previously uncharacterized activity against the target protein. The phytochemicals were collected from publicly available chemical and literature databases, including PubChem [62], ChEMBL [63], ZINC [64], as well as literature sources indexed in Google Scholar [65] and PubMed [66], along with other publicly accessible web-based repositories. Compounds were included if they were reported as plant-derived natural products with available SMILES representations; structures with invalid or unparseable SMILES and inorganic compounds were excluded. Duplicate compounds were identified and removed based on canonical SMILES generated by RDKit, ensuring structural uniqueness across the final library. The assembled library covers diverse phytochemical classes including terpenoids (23.5%), flavonoids (21.7%), alkaloids (15.9%), glycosides (13.1%), phenolic acids (1.8%), and coumarins (1.4%), among other structurally diverse natural products.

To evaluate the reliability of predictions made during virtual screening, Applicability Domain (AD) analysis was performed on the complete phytochemical library (11,032 compounds) using a leverage-based Williams plot. Leverage values were calculated from the PCA-transformed descriptor space, with the warning threshold defined as h = 3(*p* + 1)/n, where *p* is the number of principal components and n is the training set size. Compounds with leverage below h and a standardized Mahalanobis distance below 3.0 were considered within the AD of the model.

### 4.7. Drug-Likeness Filtering and Molecular Docking

To further refine the screened compounds and enhance their potential as drug candidates, Lipinski’s rule of five was applied to evaluate their drug-likeness properties. This rule assesses key physicochemical parameters, including molecular weight, lipophilicity (logP), hydrogen bond donors, and hydrogen bond acceptors, which are commonly associated with favorable oral bioavailability. The application of these criteria allowed the prioritization of phytochemicals that were not only predicted to be active by the machine learning models but also possessed desirable pharmacokinetic characteristics. However, some compounds predicted as active did not satisfy these criteria and were excluded from further analysis.

To investigate the molecular interactions between the screened phytochemicals and the target protein, a molecular docking approach was employed. The three-dimensional structure of the CLEC4M protein was retrieved from the Protein Data Bank (PDB) [67], a publicly accessible repository that provides experimentally determined high-quality three-dimensional structures of biological macromolecules, including proteins and nucleic acids [68]. The structure was selected following a systematic evaluation of all available CLEC4M crystal structures in the PDB using ERRAT scores, VERIFY3D assessment, and Ramachandran plot analysis obtained from the SAVES v6.1 server (Supplementary File S6). Before docking, the protein structure was refined using UCSF Chimera v1.19 [69]. During the preparation stage, water molecules and non-standard residues were removed, polar hydrogens were added, and the structure was energy-minimized to obtain a stable conformation suitable for docking studies. The potential ligand-binding pocket of the protein was subsequently identified using the CASTp server 3.0 [70], which analyzes protein surface topology to detect possible binding cavities [71]. Virtual screening and molecular docking of the selected compounds were carried out using PyRx (https://pyrx.sourceforge.io/) [72]. In PyRx, the docking grid was centered at coordinates X = 2.9334, Y = 25.7231, Z = 31.4971, with dimensions of X = 32.2576 Å, Y = 15.7616 Å, and Z = 25.2695 Å, fully enclosing the predicted binding cavity. An exhaustiveness value of 8 was applied to ensure thorough conformational sampling. The docking procedure evaluated the binding orientation and affinity of each ligand within the predicted active site of the protein. Compounds exhibiting lower binding energy values and favorable docking conformations were considered to have stronger binding affinity with the target protein and were selected for further analysis. Finally, the protein–ligand interactions of the best-scoring docking complexes were visualized and analyzed using BIOVIA Discovery Studio Visualizer v25.1.0.24284 [73] for two-dimensional interaction diagrams and UCSF ChimeraX v1.11.1 [74] for three-dimensional structural visualization.

### 4.8. ADMET and Pharmacokinetic Analysis

The pkCSM web server [75] was utilized to evaluate the pharmacokinetic properties, physicochemical parameters, and drug-likeness profiles of the selected ligands. pkCSM is a free computer program that uses molecular structure-based graphs to predict the Absorption, Distribution, Metabolism, Excretion, and Toxicity (ADMET) properties of possible drug candidates. The server provides predictions for several pharmacokinetic parameters, including water solubility, intestinal absorption, blood–brain barrier permeability, interactions with cytochrome P450 enzymes, and total clearance. For this analysis, the Simplified Molecular Input Line Entry System (SMILES) representations of the selected lead compounds were submitted to the pkCSM platform to estimate their pharmacokinetic behavior and assess their suitability as potential drug candidates. These predictions aided in the further prioritization of compounds with favorable ADMET profiles for subsequent analysis.

### 4.9. Density Functional Theory (DFT) Analysis

The structural and electronic properties of the target lead compounds were analyzed using density functional theory (DFT). The computational package that was used to perform all quantum chemical calculations is the Gaussian 09. Optimization of the geometry of the three chosen compounds (PubChem 42608095, PubChem 5281601 and PubChem 11243993) was done with a functional of B3LYP with the basis set of 6-311G, which is considered to be able to predict the geometry of molecules and electronic characteristics of organic molecules in a fairly reliable manner. The PubChem database was used to first obtain the molecular structures of the compounds and convert them to the corresponding input formats. The entire geometry optimization of the structures was then performed without any symmetry consideration to give the most stable conformations [76].

The Highest Occupied Molecular Orbital (HOMO) and Lowest Unoccupied Molecular Orbital (LUMO) energies were calculated by means of the Frontier Molecular Orbital (FMO) analysis. These orbitals are important in the prediction of molecules’ reactivity, capability for charge transfer, and stability. To measure the electronic stability and reactivity of the molecules studied, the HOMO–LUMO energy gap was determined. The HOMO and LUMO energies were used to compute global quantum reactivity descriptors, such as ionization potential (I), electron affinity (A), chemical potential (μ), electronegativity (χ), chemical hardness (η), chemical softness (S), electrophilicity index (ω), nucleophilicity index (N), and other electronic charges. In addition, Molecular Electrostatic Potential (MEP) surfaces were created in order to observe the distribution of charges on the surface of the molecules and to determine possible electrophilic and nucleophilic sites of attack [77].

### 4.10. Molecular Dynamics (MD) Simulations

To further evaluate the stability and dynamic behavior of the selected protein–ligand complexes, molecular dynamics (MD) simulations were performed. The simulations were carried out using the GROMACS software suite 2025. This approach allows the investigation of molecular motions and binding stability of the complexes under explicit solvent conditions [78]. The topology of the protein was generated using the CHARMM36-jul2022 force field, while ligand topology parameters were obtained through the CGenFF server [75]. The protein–ligand complexes were placed in a cubic simulation box with a minimum distance of 1.0 nm from the box edges and solvated with TIP3P water molecules. To neutralize the system, four Na^+^ counter ions were added to achieve an overall neutral charge. Prior to the production simulation, the system underwent energy minimization using the steepest descent algorithm to remove steric clashes and obtain a stable conformation. Subsequently, a two-step equilibration process was performed: the NVT ensemble (constant number of particles, volume, and temperature) was applied to stabilize the system temperature at 300 K, followed by the NPT ensemble (constant number of particles, pressure, and temperature) to maintain stable pressure conditions [79].

After equilibration, a 100 ns production MD simulation was conducted under periodic boundary conditions to mimic a realistic biological environment. The resulting trajectories were analyzed to evaluate important structural parameters, including root mean square deviation (RMSD), root mean square fluctuation (RMSF), radius of gyration (Rg), and hydrogen bonding interactions. These analyses provided insights into the conformational stability, flexibility, and binding interactions of the protein–ligand complexes throughout the simulation period [80].

### 4.11. Binding Free Energy Calculations (MM/GBSA)

To further evaluate the binding strength and energetic stability of the protein–ligand complexes, Molecular Mechanics/Generalized Born Surface Area (MM/GBSA) calculations were performed. MM/GBSA is a widely used computational approach for estimating the binding free energy of protein–ligand complexes by combining molecular mechanics energy terms with implicit solvent models. This method provides an efficient way to estimate binding affinity by considering contributions from van der Waals interactions, electrostatic interactions, and solvation effects [81].

In this study, binding free energy calculations were performed using the gmx_MMPBSA module [82]. A total of 2500 snapshots extracted from the last 25 ns of the 100 ns molecular dynamics simulation trajectory were used to ensure representative sampling of the protein–ligand interactions throughout the simulation period. The binding free energy was estimated using the following equation:

ΔG_*Bind*_ = ΔG_*complex*_ − (ΔG_*receptor*_ + ΔG_*ligand*_) (1)

ΔG*_complex_* is the free energy of the whole receptor–ligand complex, ΔG*_receptor_* is the free energy of the receptor by itself, and ΔG*_ligand_* is the free energy of the ligand by itself. The binding free energy ΔG*_Bind_* is the difference between the complex and the sum of the individual receptor and ligand energies. It measures the affinity of the ligand to the protein.

Each term in Equation (1) is calculated as

ΔG_*Bind*_ = ΔG_*elect*_ + ΔG_*vdw*_ + ΔG_*solvation*_ − TΔS (2)

In Equation (2), ΔG*_elect_* is electrostatic energy and ΔG*_vdw_* is the van der Waals energy, while ΔG*_solvation_* is the solvation energy (polar and non-polar).

By calculating ΔG*_Bind_* for each of the receptor–ligand complexes, we can calculate the relative binding affinities of different ligands to the protein, helping identify the most promising candidates for further investigation [83].

## 5. Conclusions

In this research, a comprehensive in silico approach was employed to identify potential inhibitors targeting the CLEC4M protein, a key host factor involved in Marburg virus entry. The integration of machine learning and structure-based drug design enabled efficient screening and preliminary ranking of bioactive compounds from a large phytochemical library. The Random Forest model demonstrated a superior predictive performance, enabling efficient screening of a large phytochemical library and narrowing down promising candidates. Among the screened compounds, three phytochemicals (PubChem 42608095, 5281601, and 11243993) showed computationally favorable predicted binding affinities toward CLEC4M, with favorable interactions at key active site residues. In silico ADMET predictions suggested potentially acceptable drug-like properties and safety profiles, though these require experimental confirmation. Advanced computational analyses revealed that PubChem 42608095 and 5281601 possess superior electronic properties, including lower HOMO–LUMO energy gaps, higher softness, and greater electrophilicity, suggesting possible chemical reactivity and binding potential. Molecular dynamics simulations indicated relatively stable behavior of the protein–ligand complexes. Moreover, the findings of this study are based solely on computational predictions and may serve as a preliminary framework for future research.

## Figures and Tables

**Table 1:** Dataset feature computation from SMILES using RDKit.

Features	Mean	Median	Mode	Max	Min	Std. Dev.
MolWt	320.84	270.88	226.30	827.89	127.15	131.16
MolLogP	1.31	1.57	0.02	6.23	−8.08	2.19
MaxPartialCharge	0.23	0.24	0.44	0.51	−0.01	0.09
MinPartialCharge	−0.49	−0.47	−0.37	−0.23	−0.88	0.16
MaxEStateIndex	10.94	11.79	2.79	15.10	2.79	2.69
MinEStateIndex	−1.02	−0.67	−5.40	1.06	−6.11	1.35
FpDensityMorgan1	1.28	1.29	1.50	2.00	0.53	0.23
qed	0.61	0.66	0.81	0.95	0.03	0.22
NumValenceElectrons	118.23	102.00	84.00	318.00	44.00	47.91
Chi0	15.95	13.46	10.84	43.18	5.98	6.56
Chi3n	3.93	3.62	1.83	12.91	0.74	1.78
BalabanJ	2.07	2.01	1.18	5.54	1.11	0.52
NumHeteroatoms	6.67	6.00	5.00	23.00	1.00	3.21
NumRotatableBonds	4.31	3.00	3.00	21.00	0.00	3.35
RingCount	2.57	2.00	2.00	7.00	0.00	1.25
TPSA	84.31	70.62	40.13	328.54	6.48	48.46
NumAtoms	22.04	19.00	15.00	60.00	8.00	9.17
NumHeavyAtoms	22.03	19.00	15.00	60.00	8.00	9.17
NumAromaticRings	1.51	1.00	1.00	7.00	0.00	1.19
NumSaturatedRings	0.80	1.00	0.00	7.00	0.00	0.89
NumAliphaticCarbocycles	0.38	0.00	0.00	7.00	0.00	0.73
NumAromaticCarbocycles	0.73	0.00	0.00	4.00	0.00	0.88
FractionCSP3	0.49	0.46	0.67	1.00	0.00	0.27
NumHAcceptors	4.54	4.00	4.00	16.00	0.00	2.20
NumHDonors	1.64	1.00	1.00	10.00	0.00	1.72

**Table 2:** Statistics of train and test subsets.

Subset	Active	Decoy	Total
Train	35	866	901
Test	21	366	387

**Table 3:** Performance evaluation of machine learning models on training and test data.

**Test Set**					
**Model**	**Accuracy**	**Sensitivity**	**Specificity**	**MCC**	**AUC**
**kNN**	0.9567	0.9934	0.8642	0.9077	0.8042
**SVM**	0.6915	0.3846	0.7053	0.4000	0.4201
**RF**	0.8202	0.7436	0.8240	0.8798	0.9318
**NB**	0.9567	0.9577	0.8542	0.5245	0.5645
**GB**	0.9678	0.9183	0.6282	0.5683	0.8437
**Training Set**					
**Model**	**Accuracy**	**Sensitivity**	**Specificity**	**MCC**	**AUC**
**kNN**	0.9526	0.5094	0.9231	0.4629	0.8102
**SVM**	0.7278	0.3333	0.7474	0.0393	0.6253
**RF**	0.9756	0.7692	0.9849	0.9199	0.9890
**NB**	0.9465	0.5381	0.9936	0.2964	0.5990
**GB**	0.9678	0.4564	0.9290	0.4981	0.9137

**Table 4:** Binding affinities and interacting residues of the top three compounds and 2 control drugs.

Protein	Compound Name	PubChem ID	Binding Affinity (kcal/mol)	RMSD (Å)	Interacting Residues
CLEC4M	5-hydroxy-6,7-dimethoxy-2-phenyl-2,3-dihydrochromen-4-one	42608095	−6.5	0.77	VAL 293, SER 331, LEU 333, ASN 334
	4-[(2R,3R,4S,5S)-5-(3,4-dihydroxyphenyl)-3,4-dimethyloxolan-2-yl]benzene-1,2-diol	5281601	−6.4	0.73	VAL 293, SER 331, VAL 342
	5-hydroxy-7-methoxy-2-(4-methoxyphenyl)chromen-4-one	11243993	−6.3	1.87	TRP 289, HIS 290, VAL 293, ASN 334, GLY 344
	Remdesivir	121304016	−6	0.63	HIS 290, GLN 297, GLN 302, leu 333, VAL 342
	N-(2-{[4-(dimethylamino)phenyl]amino}-4’-methyl-4,5’-bi-1,3-thiazol-2’-yl)propanamide	1117216	−5.9	2.002	TRP 289, VAL 293, TRP 341, VAL 342

**Table 5:** ADMET profiling of the top three candidate compounds.

ADMET Property	PubChem 42608095	PubChem 5281601	PubChem 11243993
Water solubility	−3.639	−3.045	−3.713
Caco2 permeability	1.449	1.001	1.203
Intestinal absorption (human)	93.879	90.461	96.134
Skin Permeability	−2.76	−2.735	−2.791
P-glycoprotein substrate	No	Yes	Yes
P-glycoprotein I inhibitor	No	No	No
P-glycoprotein II inhibitor	No	No	Yes
VDss (human)	−0.312	0.454	0.082
Fraction unbound (human)	0.022	0.119	0.164
BBB permeability	−0.243	−1.177	−0.319
CNS permeability	−2.225	−2.227	−2.103
CYP2D6 substrate	No	No	No
CYP3A4 substrate	Yes	No	No
CYP1A2 inhibitor	Yes	Yes	Yes
CYP2C19 inhibitor	Yes	No	Yes
CYP2C9 inhibitor	No	Yes	Yes
CYP2D6 inhibitor	No	No	No
CYP3A4 inhibitor	No	No	No
Total Clearance	0.145	−0.069	0.768
Renal OCT2 substrate	No	No	No
AMES toxicity	No	No	No
Max. tolerated dose (human)	0.274	0.145	0.678
hERG I inhibitor	No	No	No
hERG II inhibitor	No	Yes	Yes
Oral Rat Acute Toxicity (LD50)	2.213	1.921	2.408
Oral Rat Chronic Toxicity (LOAEL)	1.629	2.52	1.258
Hepatotoxicity	No	No	No
Skin Sensitization	No	No	No
*T. pyriformis* toxicity	0.567	0.403	0.656

**Table 6:** HOMO, LUMO, energy gap, and global chemical reactivity descriptors calculated at the DFT/B3LYP/6-311G level.

DFT Parameters	Formula	PubChem 42608095	PubChem 5281601	PubChem 11243993
Dipole moment (Debye)		1.613618	7.549542	6.043968
Electronic energy (Hartree)		−1033.498346	−1032.308641	−1074.029739
LUMO (eV)		−2.028	−2.196	−0.485
HOMO (eV)		−6.123	−6.275	−5.754
Energy gap (eV)	E_HOMO_ − E_LUMO_	4.095	4.075	5.269
Electron Affinity (A, eV)	A = −*E*_*LUMO*_	2.028	2.196	0.485
Ionization Potential (I, eV)	I = −*E*_*HOMO*_	6.123	6.275	5.754
Chemical potential (μ, eV)	μ = 1/2 (I + A)	4.076	4.236	3.120
Electronegativity (χ, eV)	*χ* = −1/2 (I + A)	−4.076	−4.236	−3.120
Chemical hardness (η, eV)	η = 1/2 (I − A)	2.047	2.039	2.634
Chemical softness (S, eV^−1^)	S = 1/η	0.489	0.491	0.380
Electrophilicity index (ω, eV)	ω = 2(μ^2^/η)	16.23	17.61	7.39
Neucleophilicity index (N, eV^−1^)	N = 1/ω	0.0616	0.0568	0.135
Additional electronic charge	= − μ/η	−54.17	−56.52	−32.24

**Table 7:** Contributions of the different energetic components (Kcal/mol) estimated using the MM-GBSA method.

Energy Components	PubChem 42608095	PubChem 5281601
**ΔEEL**	−2.88	−15.03
**ΔVDWAALS**	−16.57	−14.72
**ΔEGB**	10.82	20.81
**ΔESURF**	−2.2	−2.12
**ΔGGAS**	−19.45	−29.76
**ΔGSOLV**	8.62	18.68
**ΔTOTAL**	−10.83	−11.08

## Data Availability

The original contributions presented in this study are included in the article/Supplementary Material. Further inquiries can be directed to the corresponding author.

## Supplementary Materials

The following supporting information can be downloaded at: https://www.mdpi.com/article/10.3390/ijms27125324/s1.

## References

[1] Mire C.E., Geisbert J.B., Borisevich V., Fenton K.A., Agans K.N., Flyak A.I., Deer D.J., Steinkellner H., Bohorov O., Bohorova N. (2017). Therapeutic treatment of Marburg and Ravn virus infection in nonhuman primates with a human monoclonal antibody. Sci. Transl. Med..

[2] Anantpadma M., Kouznetsova J., Wang H., Huang R., Kolokoltsov A., Guha R., Lindstrom A.R., Shtanko O., Simeonov A., Maloney D.J. (2016). Large-scale screening and identification of novel Ebola virus and Marburg virus entry inhibitors. Antimicrob. Agents Chemother..

[3] Alsaady I.M., Bajrai L.H., Alandijany T.A., Gattan H.S., El-Daly M.M., Altwaim S.A., Alqawas R.T., Dwivedi V.D., Azhar E.I. (2023). Cheminformatics strategies unlock Marburg virus VP35 inhibitors from natural compound library. Viruses.

[4] Sadybekov A.V., Katritch V. (2023). Computational approaches streamlining drug discovery. Nature.

[5] Zameer R., Tariq S., Noreen S., Sadaqat M., Azeem F. (2022). Role of transcriptomics and artificial intelligence approaches for the selection of bioactive compounds. Drug Design Using Machine Learning.

[6] Tropsha A., Isayev O., Varnek A., Schneider G., Cherkasov A. (2024). Integrating QSAR modelling and deep learning in drug discovery: The emergence of deep QSAR. Nat. Rev. Drug Discov..

[7] Platt D., Sigamani A., Shah N. (2023). Evaluation of Complex Carbohydrates Showing Broad-Spectrum Antiviral Activity Against SARS-CoV-2, Influenza-a (H1N1) and Human Respiratory Syncytial Virus (hRSV) Strain A2 in ‘In Vitro’ Setting. https://www.preprints.org/manuscript/202309.0077.

[8] Bakheit A.H., Darwish H., Darwish I.A., Al-Ghusn A.I. (2023). Remdesivir. Profiles Drug Subst. Excip. Relat. Methodol..

[9] Borrok M.J., Kiessling L.L. (2007). Non-carbohydrate inhibitors of the lectin DC-SIGN. J. Am. Chem. Soc..

[10] Sethi A., Sanam S., Alvala M. (2021). Non-carbohydrate strategies to inhibit lectin proteins with special emphasis on galectins. Eur. J. Med. Chem..

[11] Shafiq N., Shakoor B., Yaqoob N., Parveen S., Brogi S., Mohammad Salamatullah A., Rashid M., Bourhia M. (2025). A virtual insight into mushroom secondary metabolites: 3D-QSAR, docking, pharmacophore-based analysis and molecular modeling to analyze their anti-breast cancer potential. J. Biomol. Struct. Dyn..

[12] Kokalj A. (2021). On the alleged importance of the molecular electron-donating ability and the HOMO–LUMO gap in corrosion inhibition studies. Corros. Sci..

[13] Messaoudi I., Amarasinghe G.K., Basler C.F. (2015). Filovirus pathogenesis and immune evasion: Insights from Ebola virus and Marburg virus. Nat. Rev. Microbiol..

[14] Siddig E.E., Ndembi N., Ahmed A., Muvunyi C.M. (2025). Immunogenicity, pathogenesis, and host’s immuno-responses to Marburg virus infection. Pathogens.

[15] Bhattacharyya S., Mulherkar N., Chandran K. (2012). Endocytic pathways involved in filovirus entry: Advances, implications and future directions. Viruses.

[16] Cheng H., Lear-Rooney C.M., Johansen L., Varhegyi E., Chen Z.W., Olinger G.G., Rong L. (2015). Inhibition of Ebola and Marburg virus entry by G protein-coupled receptor antagonists. J. Virol..

[17] Arnold J.N., Mitchell D.A. (2023). Tinker, Tailor, Soldier, Cell: The Role of C-Type Lectins in the Defense and Promotion of Disease. Protein Cell.

[18] Gillespie L., Gerstenberg K., Ana-Sosa-Batiz F., Parsons M.S., Farrukee R., Krabbe M., Spann K., Brooks A.G., Londrigan S.L., Reading P.C. (2016). DC-SIGN and L-SIGN Are Attachment Factors That Promote Infection of Target Cells by Human Metapneumovirus in the Presence or Absence of Cellular Glycosaminoglycans. J. Virol..

[19] Mohammed M., Alsunosi R. (2022). Effect of selecting validation dataset on building random forest and decision tree models. AlQalam J. Med. Appl. Sci..

[20] Azam S.S., Abbasi S.W. (2013). Molecular docking studies for the identification of novel melatoninergic inhibitors for acetylserotonin-O-methyltransferase using different docking routines. Theor. Biol. Med. Model..

[21] Sahu R., Mariappan R., Kumar A., Mahapatra A.K., Rajagopala S., Gupta P.K. (2025). Therapeutic potential of Andrographis paniculata against Monkeypox virus targets—A computational insight. Silico Pharmacol..

[22] Feinberg H., Mitchell D.A., Drickamer K., Weis W.I. (2001). Structural basis for selective recognition of oligosaccharides by DC-SIGN and DC-SIGNR. Science.

[23] Miller E.H., Chandran K. (2012). Filovirus entry into cells-new insights. Curr. Opin. Virol..

[24] Mumit M.A., Pal T.K., Alam M.A., Islam M.A.-A.-A.-A., Paul S., Sheikh M.C. (2020). DFT studies on vibrational and electronic spectra, HOMO–LUMO, MEP, HOMA, NBO and molecular docking analysis of benzyl-3-N-(2,4,5-trimethoxyphenylmethylene) hydrazinecarbodithioate. J. Mol. Struct..

[25] Huang Y., Rong C., Zhang R., Liu S. (2017). Evaluating frontier orbital energy and HOMO/LUMO gap with descriptors from density functional reactivity theory. J. Mol. Model..

[26] Chikalov I., Yao P., Moshkov M., Latombe J.-C. (2011). Learning probabilistic models of hydrogen bond stability from molecular dynamics simulation trajectories. BMC Bioinform..

[27] Li M.S., Klimov D., Thirumalai D. (2004). Thermal denaturation and folding rates of single domain proteins: Size matters. Polymer.

[28] Wang J., Xu M., Wang H., Zhang J. Classification of imbalanced data by using the SMOTE algorithm and locally linear embedding. Proceedings of the 2006 8th International Conference on Signal Processing.

[29] Moradi S., Nowroozi A., Nezhad M.A., Jalali P., Khosravi R., Shahlaei M. (2024). A review on description dynamics and conformational changes of proteins using combination of principal component analysis and molecular dynamics simulation. Comput. Biol. Med..

[30] Alshehri F.F. (2023). Integrated virtual screening, molecular modeling and machine learning approaches revealed potential natural inhibitors for epilepsy. Saudi Pharm. J..

[31] Samad A., Ajmal A., Mahmood A., Khurshid B., Li P., Jan S.M., Rehman A.U., He P., Abdalla A.N., Umair M. (2023). Identification of novel inhibitors for SARS-CoV-2 as therapeutic options using machine learning-based virtual screening, molecular docking and MD simulation. Front. Mol. Biosci..

[32] Aldakheel F.M., Alduraywish S.A., Dabwan K.H. (2025). Integrating machine learning driven virtual screening and molecular dynamics simulations to identify potential inhibitors targeting PARP1 against prostate cancer. Sci. Rep..

[33] Almatroudi A. (2024). Integrative Machine Learning, Virtual Screening, and Molecular Modeling for BacA-Targeted Anti-Biofilm Drug Discovery Against Staphylococcal Infections. Crystals.

[34] Liu T., Lin Y., Wen X., Jorissen R.N., Gilson M.K. (2007). BindingDB: A web-accessible database of experimentally determined protein–ligand binding affinities. Nucleic Acids Res..

[35] Mysinger M.M., Carchia M., Irwin J.J., Shoichet B.K. (2012). Directory of useful decoys, enhanced (DUD-E): Better ligands and decoys for better benchmarking. J. Med. Chem..

[36] Douzas G., Bacao F. (2026). imbalanced-learn-extra: A Python Package for Novel Oversampling Algorithms. J. Open Res. Softw..

[37] Chawla N.V., Bowyer K.W., Hall L.O., Kegelmeyer W.P. (2002). SMOTE: Synthetic minority over-sampling technique. J. Artif. Intell. Res..

[38] Jiang J., Zhang C., Ke L., Hayes N., Zhu Y., Qiu H., Zhang B., Zhou T., Wei G.-W. (2025). A review of machine learning methods for imbalanced data challenges in chemistry. Chem. Sci..

[39] McKinney W. (2011). pandas: A foundational Python library for data analysis and statistics. Python High Perform. Sci. Comput..

[40] Katubi K.M., Saqib M., Mubashir T., Tahir M.H., Halawa M.I., Akbar A., Basha B., Sulaman M., Alrowaili Z., Al-Buriahi M. (2023). Predicting the multiple parameters of organic acceptors through machine learning using RDkit descriptors: An easy and fast pipeline. Int. J. Quantum Chem..

[41] Sieg J., Feldmann C.W., Hemmerich J., Stork C., Sandfort F., Eiden P., Mathea M. (2024). MolPipeline: A python package for processing molecules with RDKit in scikit-learn. J. Chem. Inf. Model..

[42] Biessmann F., Salinas D., Schelter S., Schmidt P., Lange D. (2018). “Deep” Learning for Missing Value Imputationin Tables with Non-numerical Data. Proceedings of the 27th ACM International Conference on Information and Knowledge Management.

[43] Gupta P., Bagchi A. (2024). Data manipulation with pandas. Essentials of Python for Artificial Intelligence and Machine Learning.

[44] Gommers R., Virtanen P., Haberland M., Burovski E., Reddy T., Weckesser W., Oliphant T.E., Cournapeau D., Nelson A., Roy P. (2024). Scipy/Scipy: SciPy 1.15.0.

[45] Zollanvari A. (2023). Supervised learning in practice: The first application using scikit-learn. Machine Learning with Python: Theory and Implementation.

[46] Bahmani A., Saaidpour S., Rostami A. (2017). A simple, robust and efficient computational method for n-octanol/water partition coefficients of substituted aromatic drugs. Sci. Rep..

[47] Ivosev G., Burton L., Bonner R. (2008). Dimensionality reduction and visualization in principal component analysis. Anal. Chem..

[48] Niedoba T. (2014). Multi-parameter data visualization by means of principal component analysis (PCA) in qualitative evaluation of various coal types. Physicochem. Probl. Miner. Process..

[49] Carpenter K.A., Huang X. (2018). Machine learning-based virtual screening and its applications to Alzheimer’s drug discovery: A review. Curr. Pharm. Des..

[50] Korkmaz S., Zararsiz G., Goksuluk D. (2014). Drug/nondrug classification using support vector machines with various feature selection strategies. Comput. Methods Programs Biomed..

[51] Rigatti S.J. (2017). Random forest. J. Insur. Med..

[52] Metsis V., Androutsopoulos I., Paliouras G. Spam filtering with naive bayes-which naive bayes?. Proceedings of the CEAS.

[53] Bentéjac C., Csörgő A., Martínez-Muñoz G. (2021). A comparative analysis of gradient boosting algorithms. Artif. Intell. Rev..

[54] Konstantinov A.V., Utkin L.V. (2021). Interpretable machine learning with an ensemble of gradient boosting machines. Knowl.-Based Syst..

[55] Amer A.A., Ravana S.D., Ariyaluran Habeeb R.A. (2025). Enhanced Distance-based Weighted K-Nearest Neighbor Algorithm for Data Classification. KSII Trans. Internet Inf. Syst..

[56] Al-Mejibli I.S., Alwan J.K., Abd D.H. (2020). The effect of gamma value on support vector machine performance with different kernels. Int. J. Electr. Comput. Eng..

[57] Poona N.K., Van Niekerk A., Nadel R.L., Ismail R. (2016). Random forest (RF) wrappers for waveband selection and classification of hyperspectral data. Appl. Spectrosc..

[58] Yang H., Liu X., Song K. (2022). A novel gradient boosting regression tree technique optimized by improved sparrow search algorithm for predicting TBM penetration rate. Arab. J. Geosci..

[59] Chicco D., Jurman G. (2020). The advantages of the Matthews correlation coefficient (MCC) over F1 score and accuracy in binary classification evaluation. BMC Genom..

[60] Diallo R., Edalo C., Awe O.O. (2024). Machine learning evaluation of imbalanced health data: A comparative analysis of balanced accuracy, MCC, and F1 score. Practical Statistical Learning and Data Science Methods: Case Studies from LISA 2020 Global Network, USA.

[61] Sujon K.M., Hassan R., Choi K., Samad M.A. (2025). Accuracy, precision, recall, f1-score, or MCC? empirical evidence from advanced statistics, ML, and XAI for evaluating business predictive models. J. Big Data.

[62] Kim S., Chen J., Cheng T., Gindulyte A., He J., He S., Li Q., Shoemaker B.A., Thiessen P.A., Yu B. (2023). PubChem 2023 update. Nucleic Acids Res..

[63] Gaulton A., Hersey A., Nowotka M., Bento A.P., Chambers J., Mendez D., Mutowo P., Atkinson F., Bellis L.J., Cibrián-Uhalte E. (2017). The ChEMBL database in 2017. Nucleic Acids Res..

[64] Irwin J.J., Shoichet B.K. (2005). ZINC− a free database of commercially available compounds for virtual screening. J. Chem. Inf. Model..

[65] Noruzi A. (2005). Google Scholar: The new generation of citation indexes. Libri.

[66] Kenny T., Kemp H. (2023). Improving PubMed for the Novice at the Expense of the Expert: Surveying Librarians 3 Years Post-New PubMed. J. Maine Med. Cent..

[67] Kouranov A., Xie L., de la Cruz J., Chen L., Westbrook J., Bourne P.E., Berman H.M. (2006). The RCSB PDB information portal for structural genomics. Nucleic Acids Res..

[68] Bank P.D. (1971). Protein data bank. Nat. New Biol..

[69] Pettersen E.F., Goddard T.D., Huang C.C., Couch G.S., Greenblatt D.M., Meng E.C., Ferrin T.E. (2004). UCSF Chimera—A visualization system for exploratory research and analysis. J. Comput. Chem..

[70] Tian W., Chen C., Lei X., Zhao J., Liang J. (2018). CASTp 3.0: Computed atlas of surface topography of proteins. Nucleic Acids Res..

[71] Sadaqat M., Qasim M., ul Qamar M.T., Masoud M.S., Ashfaq U.A., Noor F., Fatima K., Allemailem K.S., Alrumaihi F., Almatroudi A. (2023). Advanced network pharmacology study reveals multi-pathway and multi-gene regulatory molecular mechanism of Bacopa monnieri in liver cancer based on data mining, molecular modeling, and microarray data analysis. Comput. Biol. Med..

[72] Fatima K., Ashfaq U.A., ul Qamar M.T., Asif M., Haque A., Qasim M., Alamri M.A., Muhseen Z.T., Noor F., Sadaqat M. (2024). Advanced network pharmacology and molecular docking-based mechanism study to explore the multi-target pharmacological mechanism of Cymbopogon citratus against Alzheimer’s disease. S. Afr. J. Bot..

[73] Studio D. (2008). Discovery studio. Accelrys [2.1].

[74] Pettersen E.F., Goddard T.D., Huang C.C., Meng E.C., Couch G.S., Croll T.I., Morris J.H., Ferrin T.E. (2021). UCSF ChimeraX: Structure visualization for researchers, educators, and developers. Protein Sci..

[75] Vanommeslaeghe K., MacKerell A.D. (2012). Automation of the CHARMM General Force Field (CGenFF) I: Bond perception and atom typing. J. Chem. Inf. Model..

[76] Subramaniyan M., Pathak M. (2025). One-pot synthesis of 2, 4, 5-triarylimidazole and phenanthro [9, 10-d] imidazole derivatives catalyzed by a new set of crystalline aluminium (III) complexes via CN bond formation. Inorganica Chim. Acta.

[77] Tahiroğlu V., Gören K., Yıldıko Ü., Bağlan M. (2024). IInvestigation, structural characterization and evaluation of the biological potency by molecular docking of amoxicillin analogue of a schiff base molecule. Int. J. Chem. Technol..

[78] Abraham M.J., Murtola T., Schulz R., Páll S., Smith J.C., Hess B., Lindahl E. (2015). GROMACS: High performance molecular simulations through multi-level parallelism from laptops to supercomputers. SoftwareX.

[79] Zhang Z., Liu C.-J., Walsh M.R., Guo G.-J. (2016). Effects of ensembles on methane hydrate nucleation kinetics. Phys. Chem. Chem. Phys..

[80] Paul L., Hamad F.B. (2025). Structure-Based Identification of Natural Inhibitors of PvFKBP35: A Molecular Docking and Dynamics Approach. Comput. Struct. Biotechnol. Rep..

[81] Du X., Li Y., Xia Y.-L., Ai S.-M., Liang J., Sang P., Ji X.-L., Liu S.-Q. (2016). Insights into protein–ligand interactions: Mechanisms, models, and methods. Int. J. Mol. Sci..

[82] Yasir M., Park J., Han E.-T., Han J.-H., Park W.S., Chun W. (2024). Investigating the inhibitory potential of flavonoids against aldose reductase: Insights from molecular docking, dynamics simulations, and gmx_MMPBSA analysis. Curr. Issues Mol. Biol..

[83] Gilson M.K., Zhou H.-X. (2007). Calculation of protein-ligand binding affinities. Annu. Rev. Biophys. Biomol. Struct..

