# Supplementary material for: AI-Driven Discovery of Prototype CLEC4M Inhibitors Targeting Marburg Virus Entry via Integrated Machine Learning and Molecular Modeling
Source: Int J Mol Sci. 2026 Jun 12;27(12):5324. doi: 10.3390/ijms27125324 (PMC13300189; doi:10.3390/ijms27125324)
Supplement: Supplementary file 1 [file ijms-27-05324-s001.zip › Supplementary Figures.pdf]

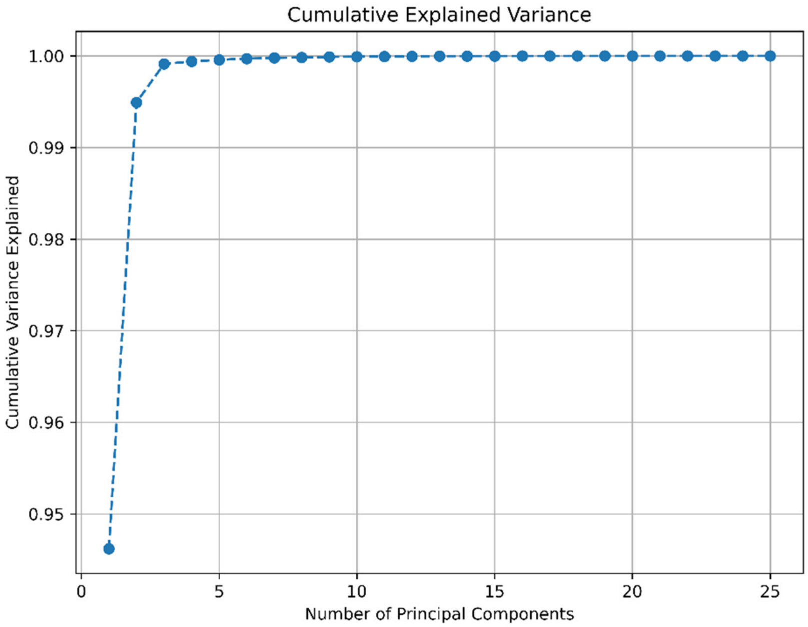

Figure S1. Cumulative variance plot showing the proportion of total variance explained by each principal component. The first principal component (PC1) accounted for 94.6% of the variance, and the second principal component (PC2) contributed 4.8%, together capturing 99.4% of the overall variance in the dataset comprising 25 molecular descriptors.

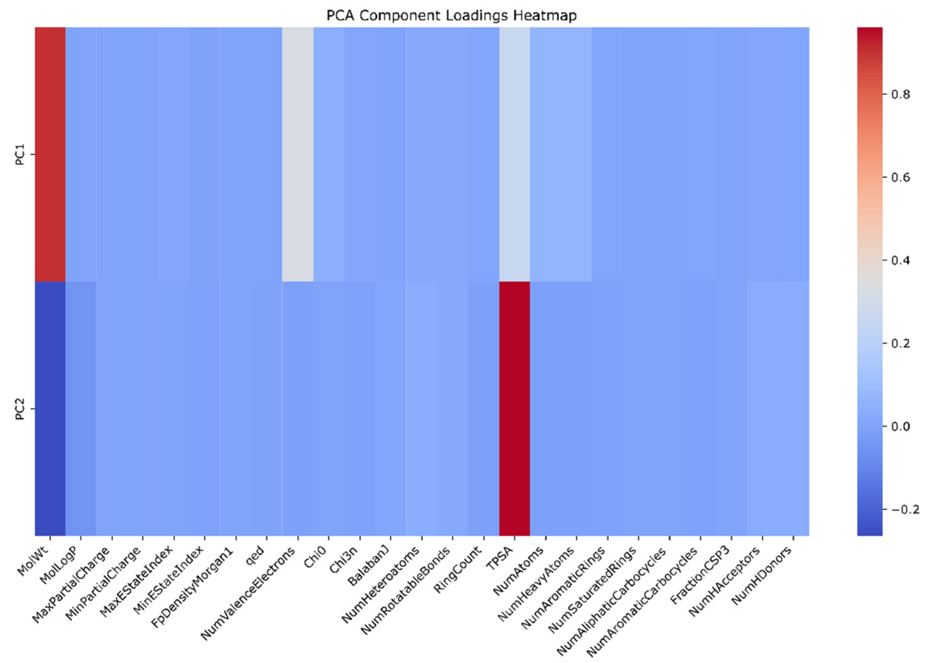

Figure S2. PCA loading heatmaps illustrating the contribution of each molecular descriptor to the first two principal components (PC1 and PC2). PC1 is predominantly driven by Molecular Weight, with secondary contributions from NumValenceElectrons and TPSA, representing the molecular size axis. PC2 is predominantly captured by TPSA contrasted against Molecular Weight, reflecting a polarity axis.

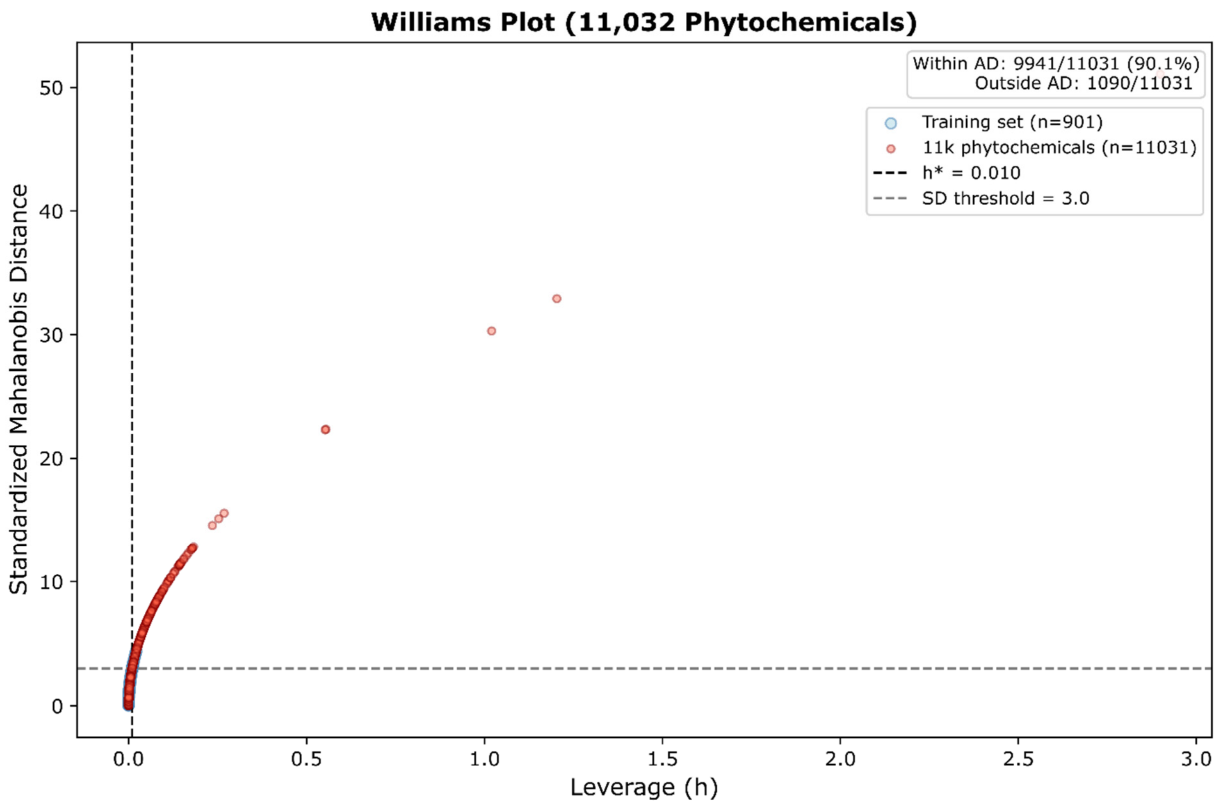

Figure S3. Williams plot depicting the applicability domain (AD) of the Random Forest (RF) model. Out of 11,032 phytochemical compounds screened, 9,941 (90.1%) fell within the defined AD boundary, confirming that the RF model's predictions are structurally reliable for the vast majority of the phytochemical library.

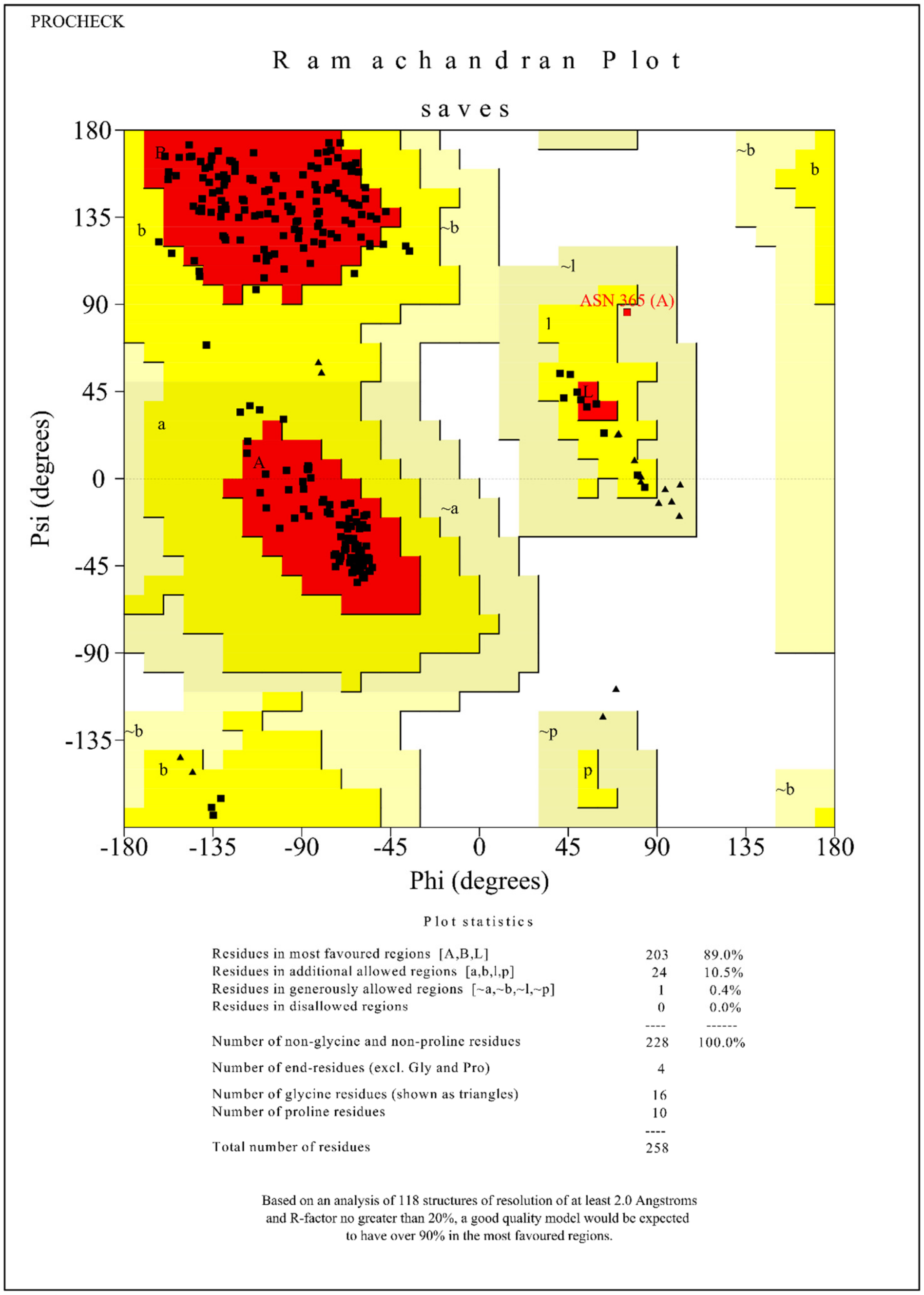

saves_01.ps

Figure S4. Ramachandran plot of the CLEC4M protein crystal structure (PDB ID: 1K9J) generated by PROCHECK, showing the distribution of backbone phi (φ) and psi (ψ) torsion angles. A total of 89.0% of residues are located in the most favoured regions [A, B, L], 10.5% in additional allowed regions, 0.4% in

generously allowed regions, and 0.0% in disallowed regions, confirming the structural reliability of the protein model.
